# A panel of DNA methylation markers for the detection of prostate cancer from FV and DRE urine DNA

**DOI:** 10.1186/s13148-018-0524-x

**Published:** 2018-07-03

**Authors:** Igor Brikun, Deborah Nusskern, Andrew Decatus, Eric Harvey, Lin Li, Diha Freije

**Affiliations:** 1 Euclid Diagnostics LLC, 9800 Connecticut Dr., Crown Point, IN 46307 USA; 2 BioStat Solutions Inc., 5280 Corporate Dr., Suite C200, Frederick, MD 21703 USA; 3 Health Decisions Inc., 2510 Meridian Parkway, Durham, NC 27713 USA; 4 Present Address: Luminex Corporation, 4088 Commercial Ave, Northbrook, IL 60062 USA

**Keywords:** Prostate cancer, DNA methylation, Urine biomarker, Liquid biopsy, Circulating DNA

## Abstract

**Background:**

Early screening for prostate cancer (PCA) remains controversial because of overdiagnosis and overtreatment of clinically insignificant cancers. Even though a number of diagnostic tests have been developed to improve on PSA testing, there remains a need for a more informative non-invasive test for PCA. The objective of this study is to identify a panel of DNA methylation markers suitable for a non-invasive diagnostic test from urine DNA collected following a digital rectal exam (DRE) and/or from first morning void (FV). A secondary objective is to determine if the cumulative methylation is indicative of biopsy findings.

**Methods:**

DRE and FV urine samples were prospectively collected from 94 patients and analyzed using 24 methylation-specific quantitative PCR assays derived from 19 CpG islands. The methylation of individual markers and various combinations of markers was compared to biopsy results. A methylation threshold for cancer classification was determined using a target specificity of 70%. The average methylation and the number of positive markers were also compared to the result of the biopsy, and the area under the receiver operating characteristic curves (AUCs) were calculated.

**Results:**

Methylation of all 19 markers was detected in FV and DRE DNAs. Combining the methylation of two or more markers improved on individual marker results. Using *6of19* methylated markers as the threshold for cancer classification yielded a specificity of 71% (95% CI, 0.57–0.86) from both DRE and FV and a sensitivity of 89% (95% CI, 0.79–0.97) from DRE and 94% (95% CI, 0.84–1.0) from FV. The negative predictive value at the 6of19 threshold was ≥ 90 for both DNA types.

**Conclusions:**

PCA-specific methylation was detected in both FV and DRE DNA. There was no significant difference in diagnostic accuracy at the 6of19 threshold between DRE and FV urine DNA. The results support the development of a non-invasive diagnostic test to reduce unnecessary biopsies in men with elevated PSA. The test can also provide patients with personalized recommendations based on their own methylation profile.

**Electronic supplementary material:**

The online version of this article (10.1186/s13148-018-0524-x) contains supplementary material, which is available to authorized users.

## Background

Prostate cancer (PCA) remains the second leading cause of death from cancer in US men even though more men die with it than because of it [1, 2]. Over 25 years of prostate-specific antigen (PSA) testing uncovered the challenges of early screening for a heterogeneous and complex disease with a highly variable natural history. Early screening with the PSA advanced the lead time of PCA diagnosis and treatment by 5 to 7 years with modest reduction in mortality observed mostly in European trials where PSA screening was not as routinely performed as in the USA [3–7]. The PSA lead time was not sufficient to significantly alter the mortality rates from prostate cancer but clinical studies showed a reduction in cancer progression for men who were screened and treated for PCA [8]. The modest benefits of early screening came at a significant cost of adverse effects and reduced quality of life [8–10]. Furthermore, PSA screening significantly increased the incidence of PCA, possibly due to the overdiagnosis of indolent tumors [2].

The majority of men diagnosed with PCA do not require treatment, but differentiating between indolent and aggressive prostate cancer remains a challenge [11]. Several novel tests aimed at diagnosing clinically significant disease have been developed including the Prostate Cancer Antigen 3 (PCA3), the 4-Kallikrein Score, SelectMDX®, ExoDX®, the Michigan Prostate Score (MiPS), Oncotype DX, and the cell cycle progression score among others [12–18]. They are performed as secondary diagnostic tests for patients undergoing PCA screening to reduce the number of biopsies and/or reduce treatment for potentially insignificant tumors. Identifying patients with high-risk disease at the time of diagnosis remains a challenge [11]. Even patients diagnosed with low-grade cancer who opt for active surveillance (AS) require continued monitoring as one third progress within 5 years and one half require intervention within 10 years [8, 19–22]. There remains a clinical need for a non-invasive prostate cancer diagnostic test to overcome the limitations of PSA and assess an individual’s risk of high-grade disease. Such a test will require a panel of cancer-specific markers that define a PCA molecular clock for pre-cancerous, indolent and potentially aggressive disease.

The hallmark of all cancers is the progressive acquisition of genomic aberrations. DNA methylation may be the most common involving hundreds if not thousands of CpG islands and can be detected in circulating DNA [23–26]. It is an ideal target for the early and non-invasive detection and monitoring of all cancers [27, 28]. Several studies have investigated the use of urine DNA methylation for PCA diagnosis using a small number of markers without achieving the accuracy needed for clinical adoption [29–32]. They also relied on a digital rectal exam (DRE) to enrich for prostate cells in urine samples, a process that is difficult to standardize. It was unclear if a DRE would be needed or if similar outcomes could be accomplished using first morning void (FV) urine samples. The advantage of using FV samples is the ability to collect multiple urine samples to reduce sampling errors associated with cell-free DNA (cfDNA) due to intra- and inter-day variation in cfDNA composition and concentration.

We undertook this study to identify a panel of markers suitable for PCA diagnosis from urine DNA and to determine if FV urine samples are an acceptable substitute for samples collected following DRE. In Brikun et al. [33], we presented evidence of extensive methylation in benign and cancerous biopsy cores of PCA patients. In the current study, we extend the methylation analysis to DNA isolated from DRE and FV urine samples.

At the start of the study, we aimed to identify a panel of markers that yields a specificity of **≥** 70% and a negative predictive value (NPV) of **≥** 90%. Prostate biopsies are an imperfect gold standard failing to diagnose up to a third of cancers on first biopsies [34, 35]. The 70% target specificity would correspond to a true specificity of over 90% had a true gold standard been available. The clinical utility and value of a urine-based PCA test depends heavily on reducing the number and cost of unnecessary biopsies, hence the target specificity and NPV. A high sensitivity would be required to achieve a negative predictive value ≥ 90%.

We selected for analysis 19 CpG islands associated with 18 genes that are methylated in prostate cancer (ADCY4, AOX1, APC, CXCL14, EPHX3, GFRA2, GSTP1, HEMK1, KIFC2, MOXD1, HOXA7, HOXB5, HOXD3 {2 islands}, HOXD9, HOXD10, NEUROG3, NODAL, and RASSF5). We developed 24 methylation-specific PCR (MS-qPCR) assays from the 19 selected markers and determined their methylation in DNA isolated from 154 urine samples obtained from 94 patients. The results show that the cumulative methylation of DRE or FV urine DNA can be used to help reduce the number of biopsies performed as a result of PSA screening. The ability to measure the methylation of a large number of markers without loss of specificity enables the development of a molecular clock for PCA to increase diagnostic lead time and to monitor disease progression in patients with potentially clinically insignificant tumors.

## Results

### Patient characteristics

Patients were classified as non-cancer if they had a negative biopsy (*n* = 52) and as cancer patients if the biopsy returned a positive finding regardless of Gleason score, the number of positive cores or volume of cancer (*n* = 42). All patients underwent transrectal ultrasound (TRUS)-guided 12-core biopsies. Patient demographics are shown in Table 1. The median Gleason score was 7 (range 1–10) and the median number of positive cores was 4 (range 1–12). Three patients who had a negative biopsy after urine collection were diagnosed with PCA within 2 years. They were included in the cancer group for the purpose of the statistical analysis.

**Table 1:** Patient demographics summarized in the overall population and by biopsy diagnosis

Variable		Cases (*n* = 42)	Controls (*n* = 52)	All (*n* = 94)
Age	*n* (%)	42 (100.0%)	50 (96.2%)	92 (97.8%)
	Median	66	64	65.5
	Mean (SD)	67.1 (7.1)	63.9 (7.6)	65.4 (7.5)
	Range	48–84	50–83	48–84
PSA	*n* (%)	40 (95%)	51 (98.1%)	91 (96.8%)
	Median	6.4	5.2	5.7
	Mean (SD)	7.1 (3.3)	5.6 (2.7)	6.3 (3.1)
	Range	3.26–18.92	0.63–14.9	0.63–18.92
Race	Alaskan Native	1 (2.4%)	0 (0.0%)	1 (1.1%)
	Asian	0 (0.0%)	1 (1.9%)	1 (1.1%)
	Black	4 (9.5%)	5 (9.6%)	9 (9.6%)
	Hispanic	1 (2.4%)	1 (1.9%)	2 (2.1%)
	White	36 (85.7%)	44 (84.6%)	80 (85.1%)
	Missing	0 (0.0%)	1 (1.9%)	1 (1.1%)
Urine samples	DRE and FV	28 (66.7%)	32 (61.5%)	60 (63.8%)
	DRE only	10 (23.8%)	17 (32.7%)	27 (28.7%)
	FV only	4 (9.5%)	3 (5.8%)	7 (7.4%)
Gleason score	*n*	39 (92.9%)	NA	
	Median	7		
	Range	6–10		
	=6	19 (45.2%)		
	7	8 (19%)		
	> 7	13 (30.9%)		
	Missing	3 (7.1%)		
Positive cores	*n*	39 (92.9%)	NA	
	Median	4		
	Range	1–12		
	≤ 3	19 (45.2%)		
	> 3	20 (47.6%)		
	Missing	3 (7.1%)		

The mean PSA for cases was calculated after excluding two outliers which were greater than two times the highest remaining PSA value from cases. All patients underwent 12-core TRUS biopsies. The number of positive cores is based on the histological examination of all 12 cores

*PSA* prostate-specific antigen, *SD* standard deviation, *DRE* digital rectal exam, *FV* first void, *NA* not applicable

### DNA methylation in DRE and FV DNA

A binary presence (> 0) or absence (=0) of methylation was used to determine the methylation status of a marker regardless of the amount of methylation detected in urine. Using a presence/absence of methylation limits any subjective interpretation of data to the analytical conditions used to assay marker methylation. Table 2 shows the estimated sensitivity and specificity of individual markers in DRE and FV DNAs. For markers with two assays, results of individual and combined assays are shown.

**Table 2:** Predictive performance of methylation status in individual assays based on urine samples from DRE (*n* = 87) and FV (*n* = 67)

Marker or assay	Sample	#Pos/#Cases	Sensitivity (95% CI)	#Neg/#Controls	Specificity (95% CI)
ADCY4	DRE	23/38	0.61 (0.45, 0.74)	38/49	0.78 (0.64, 0.87)
	FV	18/32	0.56 (0.39, 0.72)	24/35	0.69 (0.52, 0.81)
AOX1rc	DRE	27/38	0.71 (0.55, 0.83)	34/49	0.69 (0.55, 0.80)
	FV	14/32	0.44 (0.28, 0.61)	29/35	0.83 (0.67, 0.92)
APC2	DRE	10/38	0.26 (0.15, 0.42)	44/49	0.90 (0.78, 0.96)
	FV	4/32	0.13 (0.05, 0.28)	32/35	0.91 (0.78, 0.97)
CXCL14	DRE	8/38	0.21 (0.11, 0.36)	49/49	1.00 (0.93, 1.00)
	FV	9/32	0.28 (0.16, 0.45)	34/35	0.97 (0.85, 0.99)
CXCL14rc	DRE	6/38	0.16 (0.07, 0.30)	49/49	1.00 (0.93, 1.00)
	FV	4/32	0.13 (0.05, 0.28)	35/35	1.00 (0.90, 1.00)
CXCL14 Comb.	DRE	9/38	0.24 (0.13, 0.39)	49/49	1.00 (0.93, 1.00)
	FV	11/32	0.34 (0.20, 0.52)	34/35	0.97 (0.85, 0.99)
EPHX3	DRE	25/38	0.66 (0.50, 0.79)	35/49	0.71 (0.58, 0.82)
	FV	18/32	0.56 (0.39, 0.72)	24/35	0.69 (0.52, 0.81)
KIFC2	DRE	25/38	0.66 (0.50, 0.79)	38/49	0.78 (0.64, 0.87)
	FV	18/32	0.56 (0.39, 0.72)	28/35	0.80 (0.64, 0.90)
KIFC2rc	DRE	20/38	0.53 (0.37, 0.68)	42/49	0.86 (0.73, 0.93)
	FV	11/32	0.34 (0.20, 0.52)	32/35	0.91 (0.78, 0.97)
KIFC2 Comb.	DRE	30/38	0.79 (0.64, 0.89)	34/49	0.69 (0.55, 0.80)
	FV	21/32	0.66 (0.48, 0.80)	27/35	0.77 (0.61, 0.88)
GFRA2	DRE	17/38	0.45 (0.30, 0.60)	41/49	0.84 (0.71, 0.91)
	FV	13/32	0.41 (0.26, 0.58)	29/35	0.83 (0.67, 0.92)
GSTP1	DRE	18/38	0.47 (0.32, 0.63)	40/49	0.82 (0.69, 0.90)
	FV	15/32	0.47 (0.31, 0.64)	29/35	0.83 (0.67, 0.92)
HEMK1	DRE	15/38	0.39 (0.26, 0.55)	46/49	0.94 (0.83, 0.98)
	FV	8/32	0.25 (0.13, 0.42)	32/35	0.91 (0.78, 0.97)
HOXA7	DRE	32/38	0.84 (0.70, 0.93)	39/49	0.80 (0.66, 0.89)
	FV	21/32	0.66 (0.48, 0.80)	23/35	0.66 (0.49, 0.79)
HOXB5	DRE	29/38	0.76 (0.61, 0.87)	40/49	0.82 (0.69, 0.90)
	FV	23/32	0.72 (0.55, 0.84)	23/35	0.66 (0.49, 0.79)
HOXB5rc	DRE	27/38	0.71 (0.55, 0.83)	35/49	0.71 (0.58, 0.82)
	FV	22/32	0.69 (0.51, 0.82)	23/35	0.66 (0.49, 0.79)
HOXB5 Comb.	DRE	32/38	0.84 (0.70, 0.93)	29/49	0.59 (0.45, 0.72)
	FV	28/32	0.88 (0.72, 0.95)	20/35	0.57 (0.41, 0.72)
HOXD3a	DRE	19/38	0.50 (0.35, 0.65)	45/49	0.92 (0.81, 0.97)
	FV	15/32	0.47 (0.31, 0.64)	30/35	0.86 (0.71, 0.94)
HOXD3b	DRE	29/38	0.76 (0.61, 0.87)	37/49	0.76 (0.62, 0.85)
	FV	31/32	0.97 (0.84, 0.99)	21/35	0.60 (0.44, 0.74)
HOXD9	DRE	26/38	0.68 (0.53, 0.81)	29/49	0.59 (0.45, 0.72)
	FV	20/32	0.63 (0.45, 0.77)	25/35	0.71 (0.55, 0.84)
HOXD10	DRE	23/38	0.61 (0.45, 0.74)	42/49	0.86 (0.73, 0.93)
	FV	17/32	0.53 (0.36, 0.69)	27/35	0.77 (0.61, 0.88)
MOXD1	DRE	16/38	0.42 (0.28, 0.58)	41/49	0.84 (0.71, 0.91)
	FV	15/32	0.47 (0.31, 0.64)	32/35	0.91 (0.78, 0.97)
NEUROG3	DRE	14/38	0.37 (0.23, 0.53)	42/49	0.86 (0.73, 0.93)
	FV	7/32	0.22 (0.11, 0.39)	33/35	0.94 (0.81, 0.98)
NODAL	DRE	24/38	0.63 (0.47, 0.77)	40/49	0.82 (0.69, 0.90)
	FV	16/32	0.50 (0.34, 0.66)	28/35	0.80 (0.64, 0.90)
NODALrc	DRE	20/38	0.53 (0.37, 0.68)	41/49	0.84 (0.71, 0.91)
	FV	10/32	0.31 (0.18, 0.49)	28/35	0.80 (0.64, 0.90)
NODAL Comb.	DRE	30/38	0.79 (0.64, 0.89)	35/49	0.71 (0.58, 0.82)
	FV	19/32	0.59 (0.42, 0.74)	24/35	0.69 (0.52, 0.81)
RASSF5	DRE	9/38	0.24 (0.13, 0.39)	46/49	0.94 (0.83, 0.98)
	FV	9/32	0.28 (0.16, 0.45)	35/35	1.00 (0.90, 1.00)
RASSF5rc	DRE	10/38	0.26 (0.15, 0.42)	43/49	0.88 (0.76, 0.94)
	FV	11/32	0.34 (0.20, 0.52)	30/35	0.86 (0.71, 0.94)
RASSF5 Comb.	DRE	17/38	0.45 (0.30, 0.60)	40/49	0.82 (0.69, 0.90)
	FV	19/32	0.59 (0.42, 0.74)	30/35	0.86 (0.71, 0.94)

The estimated sensitivity and specificity of individual assays and combined markers. The column #Pos/#cases shows the number of positive tests from cases and the total number of biopsy positive cases analyzed. Similarly, the column #Pos/#controls yields the number of negative tests from controls and the number of biopsy negative controls

*CI* confidence interval, *rc* reverse complement, *Comb.* combined results as described in the “Results” section

Markers were recovered with variable frequencies from both DRE and FV. The observed sensitivities of individual assays ranged from 13 to 97% while specificities ranged from 57 to 100%. Combining the methylation information of markers with two assays showed improvement in sensitivity over individual assays without a significant loss in specificity. Several markers like HOXA7, HOXB5, and HOXD3b could be used individually to improve on PSA testing.

We had anticipated potentially excluding some markers due to constitutive methylation in cfDNA or equal methylation in cases and controls which would render them unsuitable for PCA diagnosis. However, none of the markers needed to be excluded. All markers were included in the statistical modeling.

### Statistical modeling to select the best diagnostic marker combinations from DRE and FV urine DNA

Statistical modeling was performed to identify the best-performing marker combinations. A summary table for two modeling approaches (logitboost and elastic net) is shown in Additional file 1: Table S1. The mean area under the receiver operating characteristic (ROC) curves (AUCs) obtained with the various modeling approaches ranged from 0.71 to 0.91. The number of markers ranged from as few as one to as many as 17. Neither the age nor PSA added significantly to the outcome of modeling.

Statistical modeling identified a large number of candidate marker panels for validation. Markers HOXA7, HOXB5, and HOXD3b showed high out of sample diagnostic capability. One or more of these three markers were included in the best-performing models. Table 3 shows the results obtained on training and test sets using select models. Models with as few as two markers and as many as all 19 showed comparable AUCs.

**Table 3:** Area under the receiver operating curves in training, test, and combined sets

	# of markers	Model	AUC/Train (25/58)^a^	AUC/Test (13/29)^a^	AUC/ALL (87)^b^
DRE	2	HOXA7, HOXB5	0.897	0.898	0.892
	3	HOXA7, HOXB5, HOXD3b	0.911	0.869	0.898
	4	HOXA7, HOXB5, HOXD3b, AOX1rc	0.915	0.875	0.91
	5	ADCY4, HOXA7, HOXB5, NODAL, MOXD1	0.89	0.909	0.9
	5	HOXA7, HOXD10, APC, GFRA2, KIFC2	0.892	0.886	0.888
	6	HOXA7, HOXB5, HOXD3b, HOXD9, GFRA2, AOX1rc	0.907	0.875	0.897
	7	HOXA7, HOXB5, HOXD3b, HOXD10, KIFC2, AOX1rc, HOXD3a	0.912	0.875	0.903
	8	ADCY4, HOXA7, HOXD3b, HOXB5, HOXD9, HOXD10, NODAL, KIFC2rc	0.891	0.847	0.876
	11	ADCY4, HOXA7, HOXB5, HOXD9, GSTP1, RASSF5, EPHX3, HEMK1rc, KIFC2, MOXD1, AOX1rc	0.916	0.949	0.925
	# of markers	Model	AUC/Train (20/45)^a^	AUC/Test (12/22)^a^	AUC/ALL (67)^b^
FV	2	HOXD3b, RASSF5	0.902	0.936	0.915
	3	HOXB5, HOXD3b, RASSF5	0.866	0.891	0.873
	3	HOXD3b, MOXD1, RASSF5	0.915	0.936	0.926
	4	ADCY4, HOXD3b, RASSF5, KIFC2	0.858	0.827	0.854
	6	HOXB5, HOXD3b, MOXD1, RASSF5, CXCL14, KIFC2	0.894	0.845	0.88
	8	ADCY4, HOXD3b, HOXD10, HOXD3a, MOXD1, RASSF5, CXCL14, GSTP1	0.902	0.882	0.895
	8	ADCY4, HOXB5, HOXD3b, HOXA7, HOXD9, HOXD3a, RASSF5, AOX1rc	0.824	0.891	0.846
	8	ADCY4, HOXA7, HOXD3b, HOXB5, HOXD9, HOXD10, NODAL, KIFC2rc	0.822	0.882	0.833

The AUCs for training and test sets as well as the combined (ALL) set for select model marker combinations and all 19 markers

^a^The numbers in the parentheses are the number of cases/total number of patients

^b^The number in the parentheses represents the combined training and test sets

**Fig. 1:**
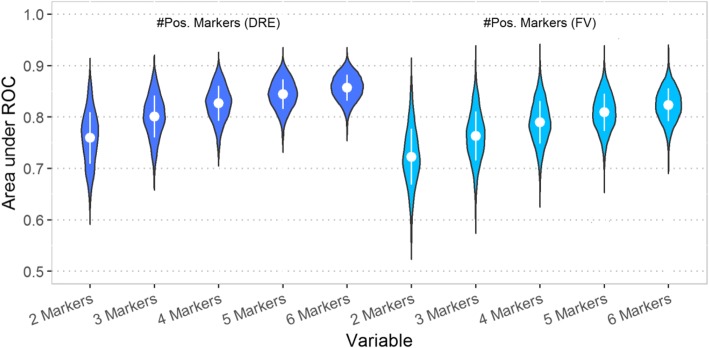
Violin plot of AUCs obtained for two, three, four, five, and six marker combinations. Violin plots of the AUCs of all two to six marker combinations using as a variable the number of positive markers. The inner part of each note shows the mean ± 1 SD. As the number of markers increases, the AUC values increase and the range of AUC values decreases

To better illustrate the number of potential smaller panels that could be derived from the 19 markers, the area under the ROC curve (AUC) of all two, three, four, five, and six marker combinations was calculated based on the number of methylated markers (> 0 methylation level). Figure 1 is a graphical representation of AUCs for all two to six marker combinations. Increasing the number of markers showed incremental improvement in overall AUC values with the six-marker combinations outperforming the ≤ 5 marker combinations. The mean AUC increased for both DRE and FV with increasing marker numbers while the range of AUC values decreased. DRE DNA methylation outperformed FV DNA methylation for the 19 markers analyzed and resulted in higher mean AUC and smaller ranges for AUC values for all two, three, four, five, and six marker combinations. However, there were many marker combinations from FV DNAs with equivalent AUCs to the best-performing combinations from DRE.

### Cumulative methylation in DRE and FV urine DNA from biopsy-positive and biopsy-negative patients

The total number of methylated markers was calculated for each DNA sample using the presence of methylation (> 0) to classify markers as positive. The median number of methylated markers in cases was 11 (range 2 to 19) in DRE and 9.5 (range 3 to 19) in FV. The median number of methylated markers in controls was 3 for both DRE and FV (range 0 to 11 for DRE and 0 to 12 for FV). Table 4 shows the sensitivity, specificity, positive predictive value (PPV), and negative predictive value (NPV) for the total number of methylated markers (*nof19*) at every threshold from 1 to 15 positive markers. For markers with two assays, only the combined data was used for the *nof19* calculations.

**Table 4:** Predictive performance of the number of positive markers among the 19 markers

Marker	Sample type	#Pos/#Cases	Sensitivity (95% CI)	#Neg/#Controls	Specificity (95% CI)	PPV (95% CI)	NPV (95% CI)
≥ 1 of 19	DRE	38/38	1.00 (1.00, 1.00)	7/49	0.14 (0.06, 0.24)	0.47 (0.45, 0.51)	1.00 (1.00, 1.00)
	FV	32/32	1.00 (1.00, 1.00)	4/35	0.11 (0.03, 0.23)	0.51 (0.48, 0.54)	1.00 (1.00, 1.00)
≥ 2 of 19	DRE	38/38	1.00 (1.00, 1.00)	15/49	0.31 (0.18, 0.43)	0.53 (0.49, 0.58)	1.00 (1.00, 1.00)
	FV	32/32	1.00 (1.00, 1.00)	8/35	0.23 (0.11, 0.37)	0.54 (0.51, 0.59)	1.00 (1.00, 1.00)
≥ 3 of 19	DRE	37/38	0.97 (0.92, 1.00)	18/49	0.37 (0.24, 0.51)	0.54 (0.49, 0.60)	0.95 (0.83, 1.00)
	FV	32/32	1.00 (1.00, 1.00)	13/35	0.37 (0.23, 0.54)	0.59 (0.54, 0.67)	1.00 (1.00, 1.00)
≥ 4 of 19	DRE	36/38	0.95 (0.87, 1.00)	25/49	0.51 (0.38, 0.65)	0.60 (0.54, 0.68)	0.93 (0.82, 1.00)
	FV	31/32	0.97 (0.91, 1.00)	19/35	0.54 (0.37, 0.71)	0.66 (0.58, 0.74)	0.95 (0.85, 1.00)
≥ 5 of 19	DRE	36/38	0.95 (0.87, 1.00)	30/49	0.61 (0.47, 0.76)	0.65 (0.58, 0.74)	0.94 (0.85, 1.00)
	FV	31/32	0.97 (0.91, 1.00)	23/35	0.66 (0.51, 0.80)	0.72 (0.64, 0.82)	0.96 (0.88, 1.00)
≥ 6 of 19	DRE	34/38	0.89 (0.79, 0.97)	35/49	0.71 (0.59, 0.86)	0.71 (0.62, 0.82)	0.90 (0.81, 0.97)
	FV	30/32	0.94 (0.84, 1.00)	25/35	0.71 (0.57, 0.86)	0.75 (0.66, 0.86)	0.93 (0.83, 1.00)
≥ 7 of 19	DRE	32/38	0.84 (0.74, 0.95)	38/49	0.78 (0.65, 0.90)	0.74 (0.65, 0.87)	0.86 (0.78, 0.95)
	FV	26/32	0.81 (0.69, 0.94)	27/35	0.77 (0.63, 0.91)	0.76 (0.66, 0.89)	0.82 (0.71, 0.93)
≥ 8 of 19	DRE	30/38	0.79 (0.66, 0.92)	43/49	0.88 (0.78, 0.96)	0.83 (0.73, 0.94)	0.84 (0.76, 0.93)
	FV	21/32	0.66 (0.50, 0.81)	30/35	0.86 (0.74, 0.97)	0.81 (0.68, 0.95)	0.73 (0.64, 0.84)
≥ 9 of 19	DRE	28/38	0.74 (0.61, 0.87)	44/49	0.90 (0.82, 0.98)	0.85 (0.74, 0.97)	0.81 (0.74, 0.90)
	FV	19/32	0.59 (0.44, 0.75)	32/35	0.91 (0.83, 1.00)	0.86 (0.74, 1.00)	0.71 (0.63, 0.81)
≥ 10 of 19	DRE	27/38	0.71 (0.58, 0.84)	46/49	0.94 (0.86, 1.00)	0.90 (0.80, 1.00)	0.81 (0.74, 0.89)
	FV	16/32	0.50 (0.34, 0.66)	33/35	0.94 (0.86, 1.00)	0.89 (0.75, 1.00)	0.67 (0.61, 0.76)
≥ 11 of 19	DRE	22/38	0.58 (0.43, 0.74)	47/49	0.96 (0.90, 1.00)	0.92 (0.81, 1.00)	0.75 (0.69, 0.83)
	FV	12/32	0.38 (0.22, 0.53)	33/35	0.94 (0.86, 1.00)	0.86 (0.69, 1.00)	0.62 (0.57, 0.69)
≥ 12 of 19	DRE	13/38	0.34 (0.21, 0.50)	49/49	1.00 (1.00, 1.00)	1.00 (1.00, 1.00)	0.66 (0.62, 0.72)
	FV	9/32	0.28 (0.16, 0.44)	33/35	0.94 (0.86, 1.00)	0.82 (0.62, 1.00)	0.59 (0.54, 0.65)
≥ 13 of 19	DRE	11/38	0.29 (0.17, 0.45)	49/49	1.00 (1.00, 1.00)	1.00 (1.00, 1.00)	0.64 (0.61, 0.70)
	FV	7/32	0.22 (0.09, 0.38)	35/35	1.00 (1.00, 1.00)	1.00 (1.00, 1.00)	0.58 (0.55, 0.64)
≥ 14 of 19	DRE	9/38	0.24 (0.13, 0.37)	49/49	1.00 (1.00, 1.00)	1.00 (1.00, 1.00)	0.63 (0.60, 0.67)
	FV	5/32	0.16 (0.06, 0.28)	35/35	1.00 (1.00, 1.00)	1.00 (1.00, 1.00)	0.56 (0.54, 0.60)
≥ 15 of 19	DRE	7/38	0.18 (0.08, 0.32)	49/49	1.00 (1.00, 1.00)	1.00 (1.00, 1.00)	0.61 (0.58, 0.65)
	FV	3/32	0.09 (0.03, 0.22)	35/35	1.00 (1.00, 1.00)	1.00 (1.00, 1.00)	0.55 (0.53, 0.58)

The calculated sensitivity and specificity, positive predictive value (PPV), and negative predictive value (NPV) associated with *n* of 19 positive markers for DRE and FV urine samples. The column #Pos/#Cases shows the number of positive cases and the total number of cases. The column #Neg/#Controls shows the number of negative controls and the total number of controls. The numbers in the parentheses show the 95% confidence interval

Using 6 out of 19 positive markers (*6of19*) as the threshold to refer a patient for a biopsy achieves the target specificity set at the start of the study. The negative predictive value for the *6of19* threshold was ≥ 0.90 for both DRE and FV with sensitivities of 0.89 for DRE and 0.94 for FV. As the methylation threshold increased, the positive predictive value of the number of methylated markers in urine DNA increased. The number of methylated markers in urine DNA can provide personalized diagnostic information that is not limited to a binary outcome to help inform subsequent clinical decisions.

**Fig. 2:**
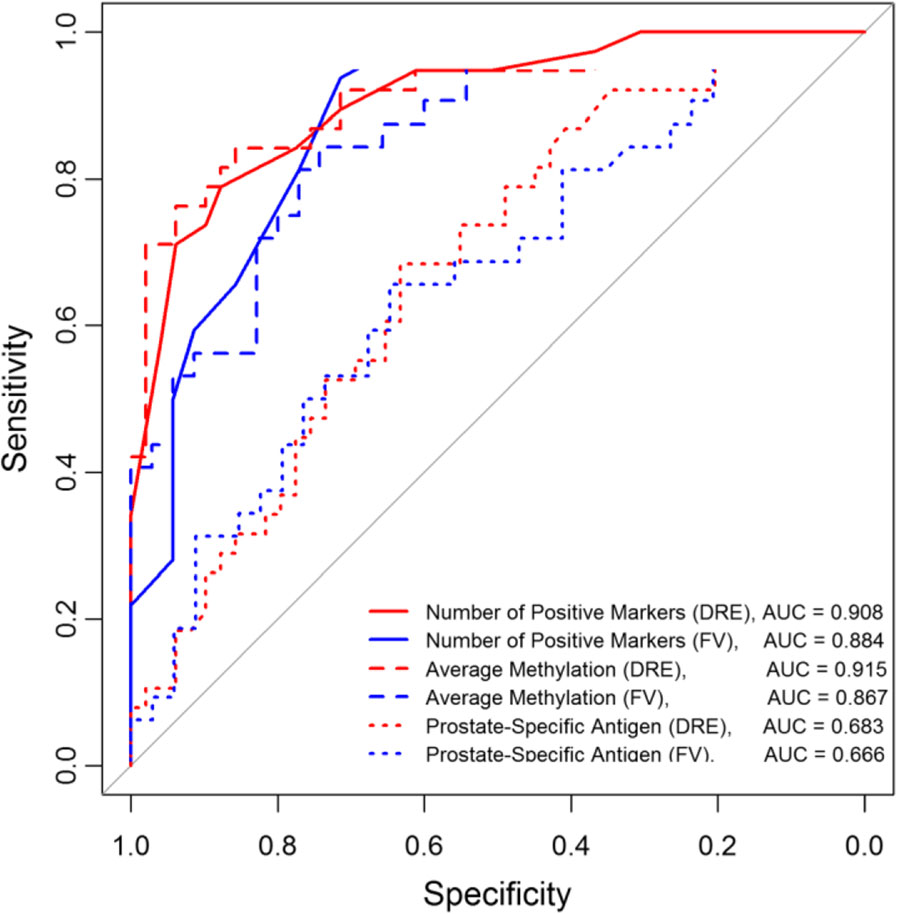
Receiver operating characteristics (ROC) curves based on the number of methylated markers and their methylation levels generated with the FV and DRE data for all 19 markers. The ROC curves obtained from DRE and FV DNAs based on the number of positive markers and the average methylation. The ROC curves for PSA were also shown for comparison

The receiver operating characteristic (ROC) curve was calculated based on the number of methylated markers and the average methylation of all 19 markers in DRE and FV DNA. Figure 2 shows the individual ROC curves for the *nof19*, and average methylation and PSA for the DRE and the FV data. Urine DNA methylation yielded AUC values ranging from 0.87 in FV to 0.92 in DRE, a significant improvement over PSA.

### Comparison of urine DNA methylation and Gleason score and tumor volume

**Fig. 3:**
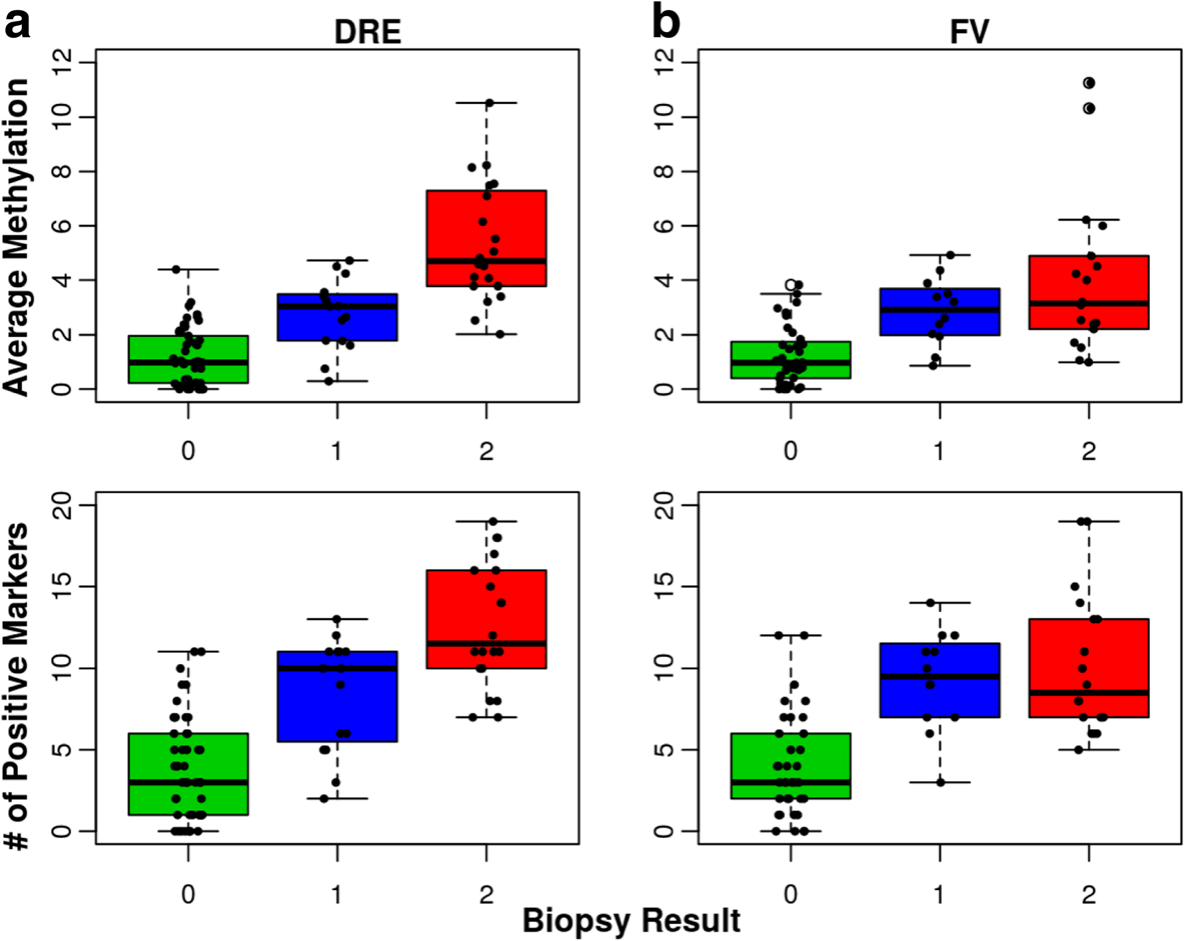
Box plot of number of methylated markers and average methylation levels. The distribution of the average methylation and the number of methylated markers in green for patients with negative biopsies (Group 0), in blue for low-risk patients (Group 1) and in red for elevated-risk patients (Group 2). **a** The results of DRE samples and **b** the results of FV samples. The line inside each box indicates the median and the lower and upper hinges correspond to the first and third quartiles. The number of patients with DRE data was 49 for Group 0 (negative biopsies), 15 for Group 1, and 20 for Group 2. The number of patients with FV data was 35 for Group 0, 12 for Group 1, and 18 for Group 2. The mean, median, 1st and 3rd quartile, and Maximum values obtained for the average methylation and the number of methylated markers are shown in Additional file 1: Table S2

The prostate cancer detected in positive biopsies (Gleason score, # of positive cores, tumor volume per core) varied widely between patients from a highly differentiated cancer focus in a single core (GS of 6, ≤1% tumor volume) to widespread, poorly differentiated cancer in multiple cores (GS of 8 to 10, up to 12 positive cores and up to 100% tumor volume per core). Similarly, the number of methylated markers and the average methylation varied widely. Patients with positive biopsies were grouped based on UCSF-CAPRA risk scoring system into a low-risk group (Group 1: CAPRA score of 1 and 2) and an elevated risk group (Group 2: CAPRA score ≥ 3) [1]. Patients in Group 2 are expected to have an intermediate risk (CAPRA score of 3–5) except for five patients diagnosed with high-grade tumors (CAPRA score 6–9). Figure 3 shows the distribution of average urine DNA methylation and the number of positive markers for all three groups.

The minimum, mean, and maximum values obtained for the number of methylated markers and average methylation for each group are shown in Additional file 1: Table S2. The mean number of methylated markers and the average methylation differed significantly between cases and controls for both DRE and first void (Wilcoxon *p* values < 0.001 for both DNA types). Furthermore, both parameters differed significantly between Group 1 and 2 patients for DRE DNA (Wilcoxon *p* values < 0.001) but not for FV DNA (Wilcoxon *p* value of 0.898 and 0.446 respectively). Pearson’s correlation coefficient between CAPRA grade and average methylation was 0.649 (95% CI, 0.403–0.808, *p* value < 0.001) for DRE DNA and 0.322 (95% CI, − 0.044–0.611, *p* value < 0.083) for FV DNA. Despite the small number of markers and the small number of patients, the correlation between grade and methylation supports further studies.

### Comparison between the DRE and FV methylation results

Paired-sample analysis was performed on the 60 samples with both DRE and FV data. Thirty-two had a negative biopsy and 28 had a positive biopsy. Given the 60 samples, the FV 6of19 threshold has a sensitivity of 0.964 (95% CI, 0.896, 1.000) and a specificity of 0.688 (95% CI, 0.527, 0.848), and the DRE test has a sensitivity of 0.929 (95% CI, 0.833, 1.000) and a specificity of 0.688 (95% CI, 0.527, 0.848). There was no statistically significant difference between the observed sensitivities and specificities of the two tests (difference in sensitivity 0.035, *p* value = 1; difference in specificity 0.000, *p* value = 1).

The paired sample analysis was also performed to compare the methylation of individual markers in DRE and FV DNAs. Table 5 shows the observed within subject mean difference in methylation levels for individual markers for the 60 patients with both DRE and FV data. The mean difference in methylation did not differ significantly between DRE and FV urine DNA for the majority of markers. Only markers AOX1, GFRA2, and NEUROG3 were better recovered from DRE samples (*p* < 0.05). The observed differences for these three markers are likely due to the position of the underlying assays within the CpG island.

**Table 5:** Paired test of within subject mean difference in methylation of individual markers observed in DRE and FV samples (*N* = 60)

Marker	Mean difference	95% CI	*p* value
Average methylation	0.356	(− 0.074, 0.786)	0.103
# of positive markers	0.667	(− 0.421, 1.754)	0.225
ADCY4	0.748	(− 0.774, 2.269)	0.329
AOX1rc	1.788	(0.479, 3.098)	0.008
APC2	0.449	(− 0.175, 1.072)	0.155
CXCL14	− 0.140	(− 0.935, 0.655)	0.725
EPHX3	0.258	(− 1.013, 1.528)	0.686
KIFC2	1.133	(− 0.161, 2.426)	0.085
GFRA2	0.766	(0.134, 1.397)	0.018
GSTP1	0.501	(− 0.203, 1.204)	0.160
HEMK1rc	− 0.131	(− 1.124, 0.861)	0.792
HOXA7	0.360	(− 0.812, 1.531)	0.542
HOXB5	− 0.823	(− 2.148, 0.502)	0.219
HOXD10	0.493	(− 0.340, 1.325)	0.241
HOXD3a	0.218	(− 0.852, 1.288)	0.685
HOXD3b	− 0.797	(− 1.602, 0.008)	0.052
HOXD9	0.909	(− 0.378, 2.196)	0.163
MOXD1	0.067	(− 0.765, 0.899)	0.873
NEUROG3	1.159	(0.202, 2.116)	0.018
NODAL	− 0.500	(− 1.508, 0.508)	0.325
RASSF5	0.312	(− 0.575, 1.199)	0.484

The mean difference in individual marker methylation between DRE and FV DNA. Only markers AOX1, GFRA2, and NEUROG3 showed statistically significant differences (*p* < 0.05)

Overall, 72% of patients had concordant diagnosis with all three tests and 82% had concordant diagnoses with FV and DRE urine methylation. Of the 28% of patients who had discordant diagnoses between the methylation results and biopsies, the majority had DNA methylation near but did not cross the threshold for one of the urine samples or had a negative biopsy when both methylation tests were above the 6of19 threshold.

## Discussion

This study shows that FV DNA methylation can be used as the basis of a non-invasive diagnostic test for PCA and yields comparable results to DRE DNA. The optimal threshold for PCA diagnosis based on target specificity ≥ 70% was *6of19* markers for both FV and DRE urine DNAs with FV slightly outperforming DRE at the 6of19 threshold. The diagnostic accuracy of a molecular test is critical when the purpose is to delay or eliminate biopsies aimed at the early diagnosis of cancer. Using the 6of19 threshold, the NPV obtained from DRE and FV was ≥ 90% with a PPV of > 70%. The urine DNA methylation test has the potential of significantly outperforming PSA and reducing the number of unnecessary biopsies.

The main challenge of using cell-free DNA (cfDNA) for diagnostic tests is the sampling error that is inherent to the DNA collection method. Genomic sequences are not equally represented in cfDNA. The use of DRE DNA was expected to reduce or eliminate the sampling error by enriching urine samples with prostate cells and/or DNA. The results of the five markers with two assays showed that interrogating the methylation of different portions of a CpG island improves the sensitivity of a marker without a significant loss of specificity from both DRE and FV DNA. The use of DRE DNA was not sufficient to overcome the sampling error for these five markers. FV DNA may be a better choice because the ease of collecting and analyzing multiple urine samples can reduce the sampling error and increase the accuracy of the results. It may also be useful to include multiple assays for each marker in clinical trials to better understand the recovery of the marker from urine DNA and to select the best-performing assays for the final test.

Paired samples analysis showed that there was no significant difference in the recovery of the majority of markers from DRE and FV DNA with the exception of AOX1, GFRA2, and NEUROG3 which were better recovered from DRE DNA. The poorer performance of these markers in FV may be a reflection of the poor representation of the assayed portion of the CpG island in FV DNA. Cancer-derived cfDNA may not be randomly fragmented and/or the stability of DNA sequences in circulation may vary leading to poorer recovery of some genomic sequences in FV DNA. The performance of these three markers in FV DNA may improve if additional assays interrogating the methylation of a different portion of the CpG island are analyzed.

Statistical modeling identified many potential models that would yield comparable results for a diagnostic test with binary outcome. Increasing the number of markers analyzed from two to six resulted in more marker combinations yielding comparable outcomes. Larger marker panels may outperform panels with two or three markers because they better compensate for the sampling errors of liquid biopsies. PSA and age were included in the statistical modeling but the small number of patients prevented meaningful correlations with methylation levels. The inclusion of the age of patients and PSA in larger studies would be important given the wide range of patients’ age, PSA levels, and biopsy results at the time of diagnosis.

How many markers are necessary for a PCA diagnostic test and which ones to choose will depend on the purpose of the test and the clinical utility and value needed to justify clinical adoption. A diagnostic test for patients with elevated PSA can easily be accomplished using 6 to 12 markers. Ideally, the panel will include markers that are indicative of the Gleason score and the tumor volume. Predictive and prognostic tests and tests to monitor the progression of cancer in patients on active surveillance or following treatment will require larger targeted panels.

The methylation of the 19 marker panel in DRE DNA was better at identifying patients with elevated risk for significant cancer (higher volumes and higher Gleason scores) than FV DNA. The potential enrichment of DRE samples with cells derived from the prostate may have improved the recovery of all 19 markers. Other markers or assays may perform better in FV DNA. Alternatively, the FV results may better reflect the steady state release of DNA from tumor cells and may provide additional information about the underlying cancer. The average methylation outperformed the number of methylated markers at differentiating between patients with low and elevated risk for significant disease. It is possible that including the level of methylation reduces potential analytical errors from incomplete DNA deamination which is inherent to the bisulfite conversion method. The analytical detection methods can be further optimized when validation studies are performed and absolute quantitation of methylation markers can be used to further improve the accuracy of the test. It is not known if the level of methylation observed for individual markers in urine DNA is directly proportional to the level of methylation observed in the prostate. Correlation of urine and biopsy DNA methylation during validation studies will help identify the most representative markers for the clinical test.

The markers used for this study were selected based on analytical conditions, i.e., they could be analyzed under the same bisulfite conditions. DNA methylation affects a large number of markers in cancer and other tissues. The recovery of cancer-specific markers from cfDNA is not well understood. It was not clear at the start of the study how well a panel of 19 DNA methylation markers would perform in FV or DRE urine. This study shows that DNA methylation could be detected in the urine of patients diagnosed with small well-differentiated tumors. It makes it likely that larger panels could be successfully analyzed and correlated to the aberrant methylation of the prostate tissue.

The true potential of using DNA methylation for a non-invasive PCA diagnostic test can be inferred from Table 4. The likelihood of a positive biopsy increases with increasing number of positive markers. The test can provide patients with personalized recommendations based on their own methylation signature. Once predictive and prognostic markers are added to the panel, the urine methylation score can be added to current PCA risk calculators. Patients with positive methylation tests may be able to delay treatment and potentially biopsies in favor of active surveillance if the methylation profile indicates insignificant tumors.

PCA3 and other PCA molecular tests can potentially reduce overtreatment of insignificant cancers. However, they are limited in their utility because they aim to identify patients with higher-grade disease. The diagnostic lead time afforded by PSA was not sufficient to eliminate PCA-specific mortality [3–8] and molecular tests that are secondary to PSA will have the same limitation. There remains a need for an early PCA diagnostic test that can increase the PSA lead time as well as measure the rate of PCA progression so only patients with fast-growing tumors are treated. The results of this study show that DNA methylation markers could potentially form the basis of such diagnostic tests. A true early PCA detection test would require markers that are methylated early during tumorigenesis. Currently, there is limited information regarding the temporal acquisition of methylation events in PCA. Extensive studies of urine, biopsy, and tumor DNAs will need to be performed in order to develop a true early PCA diagnostic panel.

The results of this study were not compared to other urine PCA methylation studies because of differences in the analytical conditions used to assay markers. To enable future comparisons, we included full details of the assays and conditions used in this study.

Urine DNA recovery varied widely between patients from as little as 25 ng to over a microgram. A minimum DNA yield will need to be established for inclusion in validation studies. Based on our results, the amount will likely be around 1.5 ng/ml of urine.

The assays developed for the 19 markers are semi-quantitative because they relied on a limited amplification of multiplexed markers for detection. The impact of PCR amplification on copy number will likely vary between markers. No exogenous or contrived DNA control can truly replicate urine DNA. External controls provide general guidelines to determine assay conditions and verify that they are performed reproducibly during data collection. Ultimately, the only true controls for assay validations are urine DNAs from the population under study. The assay validations performed on cancer cell line DNAs support their use for the urine methylation test. Additional assay validations will be needed when more accurate quantitation of markers is required. Alternative methods that do not involve PCR amplification could also be used for marker detection to further improve quantitation of DNA methylation.

## Conclusion

The study shows that the methylation of 19 CpG islands in FV and DRE urine DNA obtained from patients undergoing screening for PCA can be used to develop a non-invasive test for PCA diagnosis. Using 6 of 19 positive markers as the threshold to recommend a biopsy would reduce unnecessary biopsies performed because of elevated PSA. There was no difference in the diagnostic outcome at the 6of19 threshold between DRE and FV urine DNAs. Several markers such as HOXD3 and HOXA7 showed good diagnostic accuracy and can be used individually as secondary diagnostic tests for men referred for a biopsy. However, combining the methylation information of multiple markers improves diagnostic accuracy. Furthermore, the total number of methylated markers and the average methylation recovered from DRE urine samples differed significantly between patients with low and elevated risk for clinically significant disease.

## Methods

### Urine collection and DNA preparation

Urine samples were collected under an IRB protocol approved by Western Institutional Review Board (WIRB, study # 1139453, Puyallup, WA) from two urology clinics in Poughkeepsie, NY, and Toledo, OH. All patients signed an informed consent form prior to sample collection. Urine samples were collected prospectively from 106 patients who were recommended a prostate biopsy due to suspicion of cancer. The majority of patients had elevated PSA. Each patient was asked to provide two urine samples, one following a DRE and a second first morning void (FV) sample collected at home within 6 weeks of the DRE sample collection. Biopsy results were not available for 12 patients because they either opted not to undergo a biopsy after urine collection or the biopsy results were not available.

The urine samples were shipped to the lab without associated clinical information. The marker data was collected blindly. Urine samples were collected using the AssayAssure® urine preservative (Fisher Scientific). The volume varied between 20 and 90 ml. The entire urine sample was centrifuged at 2500 rpm for 10 min at room temperature. The sediment and the supernatant were stored separately at − 80 °C until processed. DNA was extracted from both urine fractions. Fifteen milliliters of the supernatant were concentrated using Amicon Ultra 30 15-ml columns (Millipore) to < 500 μl and mixed with a 500 μl of lysis buffer (4.0 M guanidium isocyanate, 1% triton X-100, 10 mM Tris pH 8.0, 1 mM EDTA, 10 μg per ml proteinase K), incubated at 50 °C for 1 h followed by chloroform extraction and isopropanol precipitation. The DNA was isolated from the sediment by resuspending the pellet directly in lysis buffer and following the protocol used for the supernatant extraction.

DNA was quantitated with the Qubit fluorometer (Life Technologies, Grand Island, NY) using a DNA quantitation kit (Life Technologies, Kit # Q32854). Samples with less than 20 ng of DNA were excluded from analysis. The recovery of DNA varied significantly between samples and ranged from less than 20 ng to over a microgram. For some patients, we isolated DNA from a larger volume to obtain sufficient amount for analysis. We used a fixed amount of DNA (10 ng) for each bisulfite conversion reaction regardless of yield. DNA was recovered from 87 (out of 94) DRE samples and 67 (out of 75) FV samples. The lower number of FV samples was due to poor patient compliance with the FV collection before biopsy. No urine samples were collected after the biopsy.

All DNAs were methylated in vitro at AluI and HaeIII sites according to manufacturer’s protocol (New England Biolabs) in preparation for bisulfite conversion. The cancer cell lines DU145 (ATCC HTB-81D, prostate cancer), PC3 (ATCC CRL-1435D, prostate cancer), CCRF-CEM (ATCC CCL-119, leukemia), and COLO-205 (ATCC CCL-222, colon cancer) were used as controls. DNAs and cell lines were obtained from ATCC.

### Bisulfite conversion and primary PCR amplification

The CpG island sequences are shown in Additional file 2. The assays were designed from portions of the CpG islands that allowed for the selection of two primary amplification primers, a Taqman hydrolysis probe, and at least two amplification primers. The primary amplification primers were separated by < 200 bp and preferably contained no CpGs or at most a single CpG dinucleotide. The Taqman hydrolysis probe contained at least three CpGs and the PCR amplification primers preferably contained two or more CpGs. The conditions of the bisulfite conversion and subsequent amplifications were optimized for 10 ng of input DNA. The length of the bisulfite treatment was determined blindly using a training set of 10 urine DNAs selected from the urine samples collected for this study. Three or more bisulfite time points were performed on 10 ng of the training set DNAs to select the best conditions for the deamination of individual markers as well as groups of markers. Markers were grouped into two bisulfite conditions, (14 min at 70 °C and 42 min at 80 °C) based on the results obtained with the training set. Ten nanograms of urine DNA (a mix of sediment and supernatant DNA) were used for each bisulfite reaction. The bisulfite conversion, DNA recovery, and amplification were as described [33]. The length of treatment used for the analysis of each assay is shown in Additional file 3: Table S4. DNAs were bisulfite treated in batches of 24 which included control DNAs (AluI and HaeIII methylated DNA from cancer cell lines, white blood cell DNA, and fully methylated CCL-119 DNA). Following bisulfite treatment and desulfonation, the DNA was eluted in 35 μl of water and 5 μl was used for primary amplifications. None of the cancer cell line DNAs were methylated at all markers.

To verify the recovery of DNA following bisulfite, two control assays were added to the primary amplification multiplexes, one for NSD1, an imprinted gene that is normally methylated in all DNA for the 14 min bisulfite and a second for the HOXD9 gene for the 42 min bisulfite. The imprinted promoter assay was used to verify the recovery of amplifiable DNA through all marker detection steps from urine collection to MS-qPCR amplification. The HOXD9 promoter is methylated in PCA but the unmethylated copy can still be detected after a 42 min bisulfite treatment using degenerate primers. The primers for the NSD1 gene were specific for the methylated copy. The primer sequences are shown in Additional file 3: Table S4.

### Biomarker panel

The panel of markers is composed of 19 CpG islands associated with 18 genes. The list of CpG islands and chromosomal coordinates are listed in Additional file 3: Table S3. The sequences are listed in Additional file 2. A subset of the markers were previously analyzed in prostate biopsy tissues (HOXB5, HOXD9, ADCY4, KIFC2, HEMK1, NEUROG3, CXCL14, RASSF5, GFRA2, MOXD1, APC, and GSTP1 [33]). Several markers (NODAL, HOXA7, HOXD3a, HOXD3b, and HOXD10) were selected based on the authors’ unpublished data and were methylated in 50% to over 85% of tumors. AOX1 and EPHX3 were selected based on published data [36–38]. Two CpG islands associated with the HOXD3 gene are separated by a few KB and flank a region previously associated with prostate cancer and were treated as two separate markers [38]. In total, 24 assays were analyzed from 19 CpG islands on 87 DRE and 67 FV DNAs. Two assays were generated from five markers (CXCL14, HOXB5, KIFC2, NODAL, and RASSF5), one from the forward strand and one from the reverse. Some of the marker assays described in Brikun et al. [33] were modified to shorten the amplicon when possible or were redesigned from a different portion of the CpG island or from the reverse compliment (rc) sequence if needed. All probes, primers, and assay conditions are listed in Additional file 3: Table S4.

### Assay validation and detection

DNA methylation was analyzed using nested methylation-specific quantitative PCR (MS-qPCR).

Taqman hydrolysis probes were labeled with FAM and quenched with BHQ1 (Biosearch Technologies, Petaluma, CA). Unlabeled primers were obtained from Biosearch Technologies or Eurofins Genomics. The primers selected for the multiplex amplification were neutral (no CGs) or degenerate (at CGs) and were designed to amplify all templates regardless of methylation. Primers were also degenerate at positions of in vitro methylation. The secondary MS-qPCR reactions were not multiplexed.

To validate the MS-qPCR assays, DNA from cancer cell lines, white blood cells, and CCL-119 methylated with SssI methyltransferase (NEB) were serially diluted and bisulfite converted in duplicate for 42 min at 80 °C or for 14 min at 70 °C. Input DNA ranged between 0.625 ng (~ 300 genomic copies) and 20 ng (~ 6000 copies). All DNAs were methylated in vitro using AluI and HaeIII methyl transferases (NEB) prior to deamination. The bisulfite converted DNA was first amplified with four primer multiplexes (M1, M4, M5, M6 as listed in Additional file 3: Table S4) to generate templates for the MS-qPCR as follows: 5 μl of the bisulfite-treated DNA were subjected to 23 cycles of 95 °C for 15 s, 58 °C for 45 s, 72 °C for 45 s using the manufacturer’s supplied buffer (adjusted to 2.5 mM MgCl_2_) and dNTPs, one unit of Takara Taq polymerase HS (Takara Bio), and 200 nM of each primer in the multiplex. For the imprinted gene, 50 nM of each primer was used in the primer mix. The amplified DNA was diluted with 300 μl of H_2_O. Four microliters were used as input for the nested qPCR reactions.

MS-qPCR reactions were performed in duplicate for 32 cycles using the manufacturer’s supplied buffer and dNTPs supplemented with 1.0 mM MgCl_2_ (2.5 mM total), 0.5 unit of Takara Taq polymerase HS, 0.66 μM forward primer (same orientation as the probe), 1.3 μM reverse primer and 0.5 to 1 μM of the probe (labeled with FAM, Biosearch Technologies) on an Illumina Eco qPCR Real-Time PCR system (Illumina, San Diego, CA). The reaction conditions for all assays were 32 cycles of 95 °C for 15 s, 68 °C for 20 s, and 64 °C for 20 s. Urine DNA was analyzed using the same conditions. Marker analysis was performed blindly without access to clinical data.

The limit of detection of individual assays from cancer cell line DNAs ranged between 0.625 and 2.5 ng of cancer cell line DNA. All markers failed to amplify from 10 ng of white blood cell DNA. On average, each doubling of bisulfite DNA amount resulted in a decrease of Cq value between 1 and 1.5. For this study, we did not exclude any Cq values obtained from urine DNA which means all analytical errors produced under the assay conditions used during this study were included in the data analyzed.

DNA control reactions were performed on an Eppendorf Mastercycler using 4 μl of the diluted primary multiplex PCR for 35 cycles of 95 °C for 20 s, 60 °C for 20 s, and 72 °C for 45 s using the manufacturer’s supplied buffer and dNTPs with 1.5 mM MgCl_2_, 0.5 unit of Takara Taq polymerase HS, and 1.0 mM of forward and reverse primers. The amplified DNA was separated on an acrylamide gel to verify the amplification of the control fragment.

For five CpG islands (RASSF5, NODAL, KIFC2, HOXB5 and CXCL14), two assays were developed to determine if the recovery of a marker from urine DNAs might be improved by interrogating the methylation of different portions of the CpG island. Data for these markers were merged using the highest methylation level detected when both assays were positive. All fragments of a CpG island are not necessarily recovered from urine DNA in a comparable copy number. The highest methylation level detected for each marker was used because it more accurately reflects its methylation status.

### Data collection

The data was tabulated using the Eco Study application provided with the Illumina ECO Real-Time PCR system. The range of Cq values obtained during assay optimizations was not used to eliminate Cq values higher than the limit of detection because cancer cell line DNAs are not valid controls for circulating DNA. Urine DNA differs from cancer cell line DNA in its representation, fragmentation pattern, and potentially its deamination rate. The limit of detection for marker assays will need to be calculated from urine DNA when larger studies are performed. A cutoff of 32 for the Cq was used as the upper limit for a positive reaction for all markers. A higher Cq represents a lower number of methylated copies in the sample. The data was further transformed by subtracting the Cq values from 32 (except for the 0 data points) to generate an increasing range of values from 0 (no amplification) to 15 (highest level of amplification, lowest Cq). The data was used directly for statistical analysis with no further manipulations.

### Statistical analysis

Each subject in the study had at least one type of urine sample (DRE or FV) collected. Subject characteristics were summarized within the cases and within the controls, respectively. The cases are defined as the subjects with positive diagnosis of prostate cancer based on biopsy, and the controls are defined as those with negative diagnosis. Arithmetic means or medians and standard deviations were summarized for continuous characteristics, and frequency and percentage were calculated for categorical characteristics. Characteristics such as Gleason score and positive cores are applicable only for cases and were summarized at both continuous and dichotomized levels. The following statistical analyses were performed for both DRE and FV samples (and DRE and FV combined) unless otherwise specified.

Sensitivity and specificity associated with the presence of individual methylation markers or their assays were computed using the observed proportion of individuals with positive markers conditional upon diagnosis status, and their 95% confidence intervals were also provided.

Similarly, sensitivity, specificity, negative, and positive predictive values and their 95% confidence intervals were calculated for each of the possible number of positive markers among the 19 markers and for various thresholds for the average methylation. The average methylation for each DNA sample was calculated by adding the values obtained for all 19 assays and dividing by 19. Box plots were generated by diagnosis status for both the number of positive markers and the average methylation levels of the 19 markers.

Multi-marker modeling was performed using machine learning algorithms including logit boost and elastic net [39, 40]. Methylation markers and clinical variables such as age and PSA were subject to variable selection by the algorithms. The optimal models were determined using a fivefold cross-validation approach. The top-performing models were ranked based on the area under the ROC curve (AUC) or Youden’s Index in the test sets. The average AUC in the test sets of selected top models was then reported for comparison. The AUC of the average methylation of the 19 markers was also calculated. In addition, a best subset procedure was also employed to search for top-performing models given the number of markers. A training set (approximately 2/3 of the data) and a test set were used.

The AUCs of all possible combinations of one to six markers were calculated based on the number of positive markers. Using all available data, violin plots (with mean and standard deviation) of the AUC of all combinations of two, three, four, five, or six markers were generated. ROC curves were plotted for the 19 markers based on either average methylation or the number of positive markers. ROC curve for PSA was also plotted for comparison.

The average number of positive markers by patient grading group was compared using the Wilcoxon rank-sum test. Grading groups of grade 0, 1, or 2 and the group combining grades 1 and 2 were considered. Similar analysis was performed to compare the means of average methylation by grading group.

A paired-sample analysis was performed on the samples with both DRE and FV data to compare the DRE 6of19 test and the FV 6of19 test. The difference in sensitivity, the difference in specificity, and corresponding 95% confidence intervals between the DRE and FV tests were calculated. An exact binomial test was used to test for differences in sensitivity and specificity of the two binary diagnostic tests [41].

All statistical analyses were performed using R with version 3.3.0 (https://cran.r-project.org), and the R package “pROC” with version 1.10.0 was used for AUC calculation. Additional calculations were performed using SAS 9.4.

## Additional files

Additional file 1: **Table S1.** Results of modeling using continuous and binary methylation levels. **Table S2.** the range of average methylation values and the number of methylated markers obtained from DRE and FV DNAs by grade. (DOCX 42 kb)

Additional file 2: Genomic sequences for CpG islands listed alphabetically. (DOCX 40 kb)

Additional file 3: **Table S3.** List of CpG islands included in the biomarker panel. **Table S4.** List of primers and probes. (DOCX 41 kb)

Additional file 4: The file contains the marker data. The patients were sorted and assigned new numbers that are unrelated to the alphanumeric code used as the identifier when the clinical samples were collected. (XLSX 35 kb)

## Abbreviations

ATCC: American Tissue Culture Collection
AUC: Area under the ROC curve
CpG: 5′-Deoxycytosine-phosphate-deoxyguanosine-3′
DRE: Digital rectal exam
FV: First morning void
MS-qPCR: Methylation-specific quantitative polymerase chain reaction
PCA: Prostate cancer
PSA: Prostate-specific antigen
ROC: Receiver operating characteristic
SD: Standard deviation
