# Supplementary material for: A panel of DNA methylation markers for the detection of prostate cancer from FV and DRE urine DNA
Source: Clin Epigenetics. 2018 Jul 3;10:91. doi: 10.1186/s13148-018-0524-x (PMC6029393; doi:10.1186/s13148-018-0524-x)
Supplement: Supplementary file 1 — Table S1. Results of modeling using continuous and binary methylation levels. Table S2. the range of average methylation values and the number of methylated markers obtained from DRE and FV DNAs by grade. (DOCX 42 kb) [file 13148_2018_524_MOESM1_ESM.docx]

**Additional file 1: Table S1. Results of modeling using continuous and binary methylation levels.**

Table S1 shows the mean sensitivity, specificity, AUC, # of markers and their standard deviation (SD) for various modeling approaches. The methods used were Elastic Net (EN) and logitboost (LB). The tuning methods were maximizing AUC (Max_AUC) and maximizing Youden’s Index (Max_YI). Markers with 2 assays were considered individually and combined.

| **Method** | **Tuning Method** | **Marker Type** | **Data** | **Age & PSA** | **Sens**  **Mean** | **Spec**  **Mean** | **AUC Mean** | **#**  **Markers Mean** | **Sens SD** | **Spec SD** | **AUC SD** | **#**  **Markers SD** |
| --- | --- | --- | --- | --- | --- | --- | --- | --- | --- | --- | --- | --- |
| EN | Max_AUC | Binary | FV | Yes | 0.65 | 0.88 | 0.84 | 9.6 | 0.23 | 0.19 | 0.09 | 4.16 |
| EN | Max_AUC | Binary | FV | No | 0.65 | 0.85 | 0.84 | 13.0 | 0.23 | 0.18 | 0.09 | 3.24 |
| EN | Max_AUC | Binary | DRE | Yes | 0.56 | 0.94 | 0.91 | 1.2 | 0.26 | 0.13 | 0.05 | 1.30 |
| EN | Max_AUC | Binary | DRE | No | 0.64 | 0.79 | 0.86 | 12.8 | 0.10 | 0.08 | 0.06 | 9.04 |
| EN | Max_AUC | Continuous | FV | Yes | 0.69 | 0.78 | 0.74 | 16.4 | 0.24 | 0.17 | 0.14 | 4.62 |
| EN | Max_AUC | Continuous | FV | No | 0.66 | 0.81 | 0.80 | 11.2 | 0.26 | 0.15 | 0.13 | 6.69 |
| EN | Max_AUC | Continuous | DRE | Yes | 0.64 | 0.94 | 0.91 | 5.4 | 0.25 | 0.09 | 0.06 | 6.66 |
| EN | Max_AUC | Continuous | DRE | No | 0.61 | 0.92 | 0.89 | 11.2 | 0.22 | 0.05 | 0.07 | 8.14 |
| EN | Max_YI | Binary | FV | Yes | 0.68 | 0.79 | 0.84 | 11.2 | 0.24 | 0.24 | 0.08 | 3.96 |
| EN | Max_YI | Binary | FV | No | 0.71 | 0.72 | 0.82 | 11.8 | 0.22 | 0.32 | 0.15 | 6.83 |
| EN | Max_YI | Binary | DRE | Yes | 0.64 | 0.92 | 0.91 | 2.0 | 0.17 | 0.13 | 0.05 | 0.71 |
| EN | Max_YI | Binary | DRE | No | 0.67 | 0.84 | 0.84 | 7.0 | 0.14 | 0.05 | 0.05 | 6.82 |
| EN | Max_YI | Continuous | FV | Yes | 0.73 | 0.81 | 0.76 | 13.6 | 0.19 | 0.13 | 0.16 | 6.80 |
| EN | Max_YI | Continuous | FV | No | 0.73 | 0.75 | 0.81 | 18.4 | 0.19 | 0.22 | 0.18 | 6.66 |
| EN | Max_YI | Continuous | DRE | Yes | 0.69 | 0.86 | 0.89 | 14.8 | 0.24 | 0.09 | 0.09 | 9.07 |
| EN | Max_YI | Continuous | DRE | No | 0.69 | 0.84 | 0.88 | 16.8 | 0.24 | 0.09 | 0.08 | 3.63 |
| LB | Max_AUC | Binary | FV | Yes | 0.83 | 0.56 | 0.78 | 3.6 | 0.17 | 0.17 | 0.13 | 1.52 |
| LB | Max_AUC | Binary | FV | No | 0.75 | 0.72 | 0.80 | 4.8 | 0.26 | 0.22 | 0.11 | 3.03 |
| LB | Max_AUC | Binary | DRE | Yes | 0.83 | 0.79 | 0.85 | 2.2 | 0.12 | 0.15 | 0.05 | 1.30 |
| LB | Max_AUC | Binary | DRE | No | 0.78 | 0.73 | 0.78 | 4.2 | 0.19 | 0.18 | 0.08 | 1.48 |
| LB | Max_AUC | Continuous | FV | Yes | 0.75 | 0.62 | 0.71 | 4.0 | 0.16 | 0.23 | 0.08 | 1.00 |
| LB | Max_AUC | Continuous | FV | No | 0.76 | 0.61 | 0.72 | 4.0 | 0.19 | 0.25 | 0.16 | 2.35 |
| LB | Max_AUC | Continuous | DRE | Yes | 0.70 | 0.84 | 0.81 | 2.2 | 0.12 | 0.11 | 0.07 | 1.30 |
| LB | Max_AUC | Continuous | DRE | No | 0.80 | 0.75 | 0.84 | 3.4 | 0.19 | 0.20 | 0.10 | 1.34 |
| LB | Max_YI | Binary | FV | Yes | 0.85 | 0.53 | 0.79 | 3.0 | 0.16 | 0.16 | 0.15 | 1.73 |
| LB | Max_YI | Binary | FV | No | 0.76 | 0.60 | 0.75 | 2.6 | 0.19 | 0.27 | 0.16 | 1.34 |
| LB | Max_YI | Binary | DRE | Yes | 0.80 | 0.74 | 0.84 | 1.0 | 0.13 | 0.11 | 0.07 | 0.71 |
| LB | Max_YI | Binary | DRE | No | 0.86 | 0.69 | 0.81 | 2.8 | 0.14 | 0.12 | 0.11 | 1.79 |
| LB | Max_YI | Continuous | FV | Yes | 0.82 | 0.62 | 0.73 | 2.6 | 0.19 | 0.17 | 0.12 | 1.14 |
| LB | Max_YI | Continuous | FV | No | 0.89 | 0.56 | 0.72 | 2.8 | 0.10 | 0.21 | 0.15 | 1.30 |
| LB | Max_YI | Continuous | DRE | Yes | 0.80 | 0.73 | 0.83 | 2.2 | 0.13 | 0.18 | 0.07 | 1.79 |
| LB | Max_YI | Continuous | DRE | No | 0.86 | 0.78 | 0.87 | 2.2 | 0.14 | 0.19 | 0.09 | 0.45 |

**Additional file 1: Table S2. the range of average methylation values and the number of methylated markers obtained from DRE and FV DNAs by grade.**

Table S2 shows the mean, median, 1^st^ and 3^rd^ quartile, and Maximum values obtained for the average methylation and the number of methylated markers for 3 classes of patients: negative biopsy (0), low risk patients (1), and elevated risk patients (2) as graded using CAPRA risk assessment criteria (as described in the manuscript). Qu=quartile.

|  | Sample Type | Biopsy Result | N | Min | 1st Qu | Median | Mean | 3rd Qu | Max |
| --- | --- | --- | --- | --- | --- | --- | --- | --- | --- |
| **# of Positive Markers** | DRE | 0 | 49 | 0 | 1 | 3 | 3.9 | 6 | 11 |
|  | DRE | 1 | 15 | 2 | 5.5 | 10 | 8.33 | 11 | 13 |
|  | DRE | 2 | 20 | 7 | 10 | 11.5 | 12.65 | 16 | 19 |
|  | DRE | 1+2 | 35 | 2 | 8 | 11 | 10.8 | 13.5 | 19 |
|  | FV | 0 | 35 | 0 | 2 | 3 | 4 | 6 | 12 |
|  | FV | 1 | 12 | 3 | 7 | 9.5 | 9.25 | 11.25 | 14 |
|  | FV | 2 | 18 | 5 | 7 | 8.5 | 10.17 | 13 | 19 |
|  | FV | 1+2 | 30 | 3 | 7 | 9.0 | 9.8 | 12 | 19 |
| **Average Methylation** | DRE | 0 | 49 | 0 | 0.22 | 0.98 | 1.2 | 1.96 | 4.39 |
|  | DRE | 1 | 15 | 0.29 | 1.79 | 3.05 | 2.75 | 3.50 | 4.72 |
|  | DRE | 2 | 20 | 2.02 | 3.79 | 4.70 | 5.33 | 7.20 | 10.53 |
|  | DRE | 1+2 | 35 | 0.29 | 2.86 | 3.79 | 4.23 | 4.93 | 10.53 |
|  | FV | 0 | 35 | 0 | 0.4 | 0.97 | 1.24 | 1.75 | 3.83 |
|  | FV | 1 | 12 | 0.86 | 2.01 | 2.92 | 2.86 | 3.61 | 4.92 |
|  | FV | 2 | 18 | 0.99 | 2.26 | 3.16 | 4.03 | 4.8 | 11.25 |
|  | FV | 1+2 | 30 | 0.86 | 2.08 | 3.16 | 3.56 | 4.33 | 11.25 |
