# Supplementary material for: A panel of DNA methylation markers for the detection of prostate cancer from FV and DRE urine DNA
Source: Clin Epigenetics. 2018 Jul 3;10:91. doi: 10.1186/s13148-018-0524-x (PMC6029393; doi:10.1186/s13148-018-0524-x)
Supplement: Supplementary file 2 — Genomic sequences for CpG islands listed alphabetically. (DOCX 40 kb) [file 13148_2018_524_MOESM2_ESM.docx]

**Genomic Sequences for CpG islands listed alphabetically.**

**ADCY4_CpGLength:1050**

TGCAGTTCCTAGCCAGGCTCAATGGTCCTTTCTTTCTCTGCTCCCAGCAACGAAAACACATCGAAAAGAAGACCCCACAAACCCCAACCCCGAAAATGCTCCCCAGGTAGAGACCCTCCCGCAGCAGAGGCTCGGCTCACCCTGCCGCTGGCCCAGGCCACTGCGAGCAGCGCCGCGAGCGCACAGAGCACGATCCCCAGCAGCAGCAGCAGCAGCGGGTACTGCTGGCTCAGGCTGTAGTAGGTCTCGTAGAAGAGGTCTTCGCTGGGGGGCGGCCGGGGGCTGAAGAGGCGGGCCATGATCTCCCCAGCCCCGAGCCCCGGGGCTGGCTAGGGCCGGGCGCCGGGTTACCTCCTTCGGCCCGGCGGGCCCCACCTGAGCTTTTCTCACCCGCTCAAAGCCGCTACCACCCCGCGCCCCCAACCTCGTGGCAATCCCGTCTCCTTTTTCAGGCCCTCCCTGCGGCCTCCCAGCCCGCTCCCAGCTGGCGATGAGGGGATCCCTCAGTCCTTTCCTCCTCCTCCCTCAAACCCGGATTGTAGAGGTGCCCTAGAAAAGCCTCATCGCCAGGAGGGCAGGATTTGGGGTTGGTGCGAGGGAGCCCCGGGTTCCCCTGATCCCCGAACTCACTGCCCGCGGCGCTGGCGCAGCGGAGTGGGCTAGGTCCGGGGAGGGAGCCGGGGACCTGATGGCGGAGTCACGCTCGCCGCTGCGCTCTGGCTCAGAGTCCCGGGCGACAGGCGCTTCCCTCCCGGCGCCGCGGCCCCCTCCCACTTCCCCGACGGGACAGCCCGGCCCCTTCCTGTGCCAAAGCCGGACTCCCCTGGTCTCCTCCCGCACTTGGTTTTGTCTCTCCTAACACCATCGCCTCCCCGCTTCAGCCCCTTCAGGGAAGGGCAATGTGGACCCTGGGAGAGATGCGAAGGGGTGGGCTGGGGTGGACCCAATGGGATGTGTCCAAATGGAGGTGAACGTTTGGTAGTGGGGAGACCGAGGGCGGGGGGCTGAAGCCTGGCGAGAATGTAGGGAAGAATGTGGGTCCTGGTGGGG

**AOX1_CpGLength:800**

GGTTGGAGGGGAGTTACTAATGTTCCCAGACTTAAATCCAGCTGGAACACCACCTAAAATATGCAGTAACATAAGACCATCAAAAGCAATGTCCCAGGACTTACAATGTTTGCTAAGACGCAAGAGGGTGTGACACAGACGCTAAGCGCCACTGGCGAGGAGATGAAGGGGTCGTCTTCATCTTCGCCGGATGATTTCCGCCCACATAGAGGGCGCCAGTGACGCCCACACACGTGCTGGTGTCCCGGGAAGAGTTCCTGGCAAAGAGCTCAGGAACGTTGGATCTTAATCAAGGCTTTCTCCGTCGGGGTGGATGGGTTGGACTTTAGGCTCCAGCAAGCCCCGCCCCACTCGGCGGGTCGGTGCCGCCGGGTCCCAGGTGCCCGCTACTTCCCAGAACCTCCGCCTCCCGCTCCGGGCCCTCGAACCAGCGCGGACACCACAATGGACCGGGCGTCCGAGCTGCTCTTCTACGTGAACGGCCGCAAGGTGAGCGCCCGCGGGCTTCCTCTGCCCCCAGACCTGCGGCCAGGGCCGGGGCAGAGAGGAGCCCCTGCCGTTCGTCCCATCCTTTCGTGCCCGCCGTTTAAGGCACTCAGGCACGGACTGGCTTTCTCCCGTAACAGCGGCTTTGCCTTCGCATTCCCACCCCTGCCTCCGGGGCAGCTAAAGGCTGCGTTTTCAGGGACTGTCCTGGATCTTTGGGCCCCCACCTTCCTTATGTTCCCCTTTCCTCTCCTTCCAAGGCTCACAAGCTGATTTCCAACCACACGCCCTCCCTAAACAGTAAGATAAAAAGT

**APC_CpGLength:550**

CAGTGACTTGTAATGTAAAATTATTCATTGTAATTCATTTAATATTATTGTTTCTCTGTGCTGCAAAAATCATAGCAATCGAGATGTAATTTATTACTCTCCCTCCCACCTCCGGCATCTTGTGCTAATCCTTCTGCCCTGCGGACCTCCCCCGACTCTTTACTATGCGTGTCAACTGCCATCAACTTCCTTGCTTGCTGGGGACTGGGGCCGCGAGGGCATACCCCCGAGGGGTACGGGGCTAGGGCTAGGCAGGCTGTGCGGTTGGGCGGGGCCCTGTGCCCCACTGCGGAGTGCGGGTCGGGAAGCGGAGAGAGAAGCAGCTGTGTAATCCGCTGGATGCGGACCAGGGCGCTCCCCATTCCCGTCGGGAGCCCGCCGATTGGCTGGGTGTGGGCGCACGTGACCGACATGTGGCTGTATTGGTGCAGCCCGCCAGGGTGTCACTGGAGACAGAATGGAGGTGCTGCCGGACTCGGAAATGGGGTAGGTGCTGGAGCCACCATGGCCAGGCTTGCTGCGGGGGGAGGGGGGAAGGTGGTTTTCCCTC

**CXCL14_|CpGLength:850**

CCTCGCAGTGCGGGTACTTTGGCTTCATTTCCAGCTTCTTCACGTCGCTGTAGCGGATCTTGGGTCCCTTCCGGGAGCACTTGCATTTGGACCCTGCGAGCGAGCGCGGGGCAACGGCTTAGTTGCTAGGCGGTCTCCTGCCCCTCGACCACCTTGGTGCCCACCCAGACCACCCCCCGCGGGATCCCAGGATGCCTAGAAATTGGCGCTTGGGTTCCCCAGGACAGGACAAGACGAGACGGCGACAAGGGGAGCTCCCCGCACTCACCGTCCACACGCGCGGTGTACAGCGCCAGCAGCAGCAGGAGCAGCGCGGCCGCCAGGAGCCTCATGCTGACCGGAGGGGCGCGGCGTGGGAGCAGGGACATGGGGAGGGCGCTGGCCCGTCGGAGCGGCGGCCCGGAGACGCCACCCAGCTCTGCTCGGCTTTCTCTGCCCGGGGCGCGCCTTCCGGCTCTGCTGGCTCCGGCTGCGCCGTCGGTGGATGCCCAGGGCTGTCTGTGGCCGTGCGCTGCGCTCTGCGCTTGTCTCCGCGCTCTCTCCACAGCCTCCCTCCGCCCGCCCTGGCCTCTTTTAAATCCGCTCCTGCCCTCGCAGCGAGCGAGCTCATTAATATGCAGAACCACTCGGTGACTCACTGAGATTTCTCAATGTGGTGAGGGGAGGAGACCTTCCCAGCCCGCCCGTCGCCCGCCCCGGGAAAGGACCCGCTATCGCAGCGGCGCACACCCGGAGCCACGCGCGCACACGCACACACACATCCGCTACCCTCTCGCTCACACAACACGCCGCCTTCGCACACTTGCACACCCATACCACAGCGCGCACACATTTCCAGCGTCCCGCTCTGCCTTTGGGGAGCCCAGACGGTGGAGAGGCTGCTGAAGGGCACTGCTTTTCTGAATCGTGTGGTTCTCTCTCTGGCCCGGCAGGCCAGCTTTTCCGAGCCGGCGGGAGACCCAGGCCCAGGGCTCAGATCCTCAAAGACCTGAAAGGGTTTTGGAGCAGGTGCCAAGTGTGAGTGCCTGCAGGCCTGACCCCCAAGGGCCC

**EPHX3_CpGLength: 2340**

CGCTCTGTCGCCCTGGCTGGAGTGCAGTGGCGCAATCTCAGCTCACTGCAACCTCCGCCTCCTGGGTTCACGCCGTTCTCCTGCCTCAGCCTCCCTAGTAGCTGGGACTACAGGCGCCCGCCACCACGCCTGGCTAATTTTTTGTATTTTTAGTAGAGACAGGGTTTCACCATGTTAGGATGGTCTCGATCTCCTGACCTCGTGATCCGCCCGCCTCGGCCTCCCAAAGTGCTGGGATTACAGGCGTGAGCCACCGCGCCTGGCCAGAAGCTGGCTTCTTGCAGGGACAGCAGGGCCGCAAGGCTCTGTGACTCTAGGCCCCATCACACCCGGCCCTGTCCCACTTCCTCTTTCGGCCAGAACCCCAGAACTCCAGTCATTCATAGGCCGGGTCCTCCTCGGGGGAACCAGGCCTGCCCCCTAGGAATCAGGGTATCCACGCCTCCTCGGCCCGCTCTCGGAGCCTGGGCCTCAACACCTGCAGATTGGCTCCCGGGCCAGGACCTGTCGCTTGAACATCAGTGGCCGCCGCCGTCCAATCACAGACGTACAGGGGCGGGCTGGCGGGCCTGTTGGGCCAGCAGGAGGACGGCGCCGGGCCGAGGCCGCTGGACCCGCCGTTGGAACCACAGCTGGGTGAAGTCAGTGCTGCTAGGTCTTTGGAGCCAGACCGAAGGGAGGGTCGCGCGCCCCTGGGTGGGTAGCACCAGGCGGCGGCCGTCGGCCGGGCCTCCCGAAGCCTCCTCCAGCCGCTAATACCGCCCGCGCCTCCCTCCTAGTCCTAGACCTGGCATTGCCTGTCCCTCTTCTTCGTCTTCCAGACCCTCCTGACCCGCCATATTAGGTCCCGGCCCCGCCCTCCGTCTCATTGCCCATTGCCCACCCCACCCCACCGCCCCCTCCCCGGCCTACCGTCAGCTCATTGGCCCAGGCTCCCGCCTTCTTCGCCCAATGTTAGGCCTCACCTCCTCTAGGATCCACCCTCTCCCCGCCCACCCGCATAAGCCAGGCCCACCATTCCAACTCTTTCTCCTTTTTGGCTTGTAGGCCGCACCTACTCTGCATATGCCCCGCCCCTCACTCCAGGTGTCGCCCACTTAACTGAAGATCCAGGATCCCCTCCTTAAGGTTTGTCTGGGCTCGGAATTGCCTTTTTTTTTTTTCTTTTTTGAGACGGAGTTTCCCTGTTGTGACCCAGGCTGGAGTACAGTGGCGCGATCTCAGCTCACTGCAACCTCCACGTCCCGGGTTCAAGCGATTCTCCTGCCTCAGCCTCCCAAGTAGCTGGGACTACAGGCGCCCGCCACCACGCCCAGCTAATTTTTGTATTTTTAGTAGAGACGGGGTTTCACCACGTTGCCCAGGCTGGTCTCGAGCTCTTGATCTCAGGTGATCCGCCCGCCTCGGCCTACCAAAGTGCTGGGATTACAGGCGTGAGCCACCGCACCCGGCCCGGAATTGCCGCCTTTAGACCCTCACCCACCCGCTGCTGAGTCTTCCTGGGCCCCGCCCCGGCCCTAAGCCCCGCACAGGACCTGCTAGGCCCCGCCCATTTATTTCCCCCCTTTCCAACCTCTTCCTCCCCAGCCTCCGCACCCTACCCTTGTTTCCTGTCCCTGTCGCGCCCAGGTGTTTACCTGGCACTCAGGTGAGTGGTGCGCTCTGGCTGTTTTCTGTCGGAGCCGCCCGCCTCTTCCTTCAGCGCGTCCCACAAATCCCGACGGCACGGAGGGGCCCCAGGCCAAGGGCGATGGGCCCCTGAGCCCTGACACCGCTTCGCCGCTGCTGCAGGTGCCCCTGGCCGGCAGCGCCGCCGTGGTCCCGGAGCGCGGCGACATGCCGGAGCTGGTGGTGACCGCGCTGCTGGCGCCGTCGCGCCTGTCGCTGAAGCTGCTGCGCGCCTTCATGTGGAGCCTGGTGTTCTCGGTGGCGCTGGTGGCCGCGGCGGTCTACGGCTGCATAGCGCTCACGCACGTGCTGTGCCGGCCCCGGCGCGGCTGCTGCGGGCGCCGTCGGAGCGCGTCCCCCGCCTGCCTGAGCGACCCCTCGCTGGGTGAGCACGGTTTCCTGAACCTCAAGGTGATCGCACGGGGGCGCTATCGGGGCTGCGGGGTCGCGTGGGGTCGAGACGGTTCAAGCCTCGGGAGAGAGGGTTCCCCAGGCTTCAGGCTGTGCCCTCCCCCTAGAGCTCGGGCCTGCGTCTGCACTATGTCTCGGCTGGACGAGGTAACGGACCCCTCATGCTGTTTCTGCACGGCTTCCCTGAGAACTGGTACGTCTGGGGCCAGGGGCCTGAGGCCCACGGGACTCGGGGGCAGGTGGAGCTTGGGCTCCCAAGAG

**GFRA2_CpGLength:1800**

CTAGCATTTACTGGATTCCAGAGTCTTGTTATTTAAGAATGCATCTTAAACGGTACTATCAAATTCATGTTACGTGCAGCCCAGATTGTTTTGGGCAGCACGAAAAGTTTCTGAGGCGCTGCGTGTACCCCACCCCAGGACACCGTGTGTGCGCGCCGAGCTGAGTGCGAGGAACGTGGCGCGAGGGCCGGGGGATGCCGGGCTGCGTGGGTGTGAGCCCTCGCGCGACCGCGACCCCGCGCCTCTCCCGCTCTCGCCGGAACGTGACCGCAGCCGCACCTCTCCTCCAGCCCTTTCCCAGCCAGACGCTTCCTTTTAGGTCCTTCTGGGCGTTTATTGTAAATTCTGCGACTAAAACACGCCGGTGAGCCCGGCCCACCGACAGATGGATCAATCGCCCCCTTCCCGGCTAGGGGAGGAGGAACCCCCCAACCCCGGAGCCTAGGGAGCCGGGAGCTGCCTCGGGACGAGCTCCTCGGAGCCCAGCCGGCTGCGGAGCCCCGGCCCGGGTCGGTCTCGGGGCCCTCCTGCCGGGGTGGGGTGCGAGCCCCTGCCCGATTCCTCTGGGGCGGTTCAGGCAGGTTTGCCGGCCTCCGAGGAGGTGGTCAGGGCGCCCTGGCCCAGCAGGCTTCTTCCCGAGCCGGGGGGAGGGGAGACCGGCTGGGGAAGGGGCATCTCGAAGGGGTGGAGGCCGGGGCGGGCGGGAGGCAAGCGCGCCGCGGGCGTGAGGGCAAAGTTCCCGAGGTCCGCGCGGAGAGCACACGTGTATGTGCGCGCGGGGCTAGGCCGGGGCCGGCAGGATGCGTTGGGTTCGGGGGCGCGCGGGGCCGGCGCCGAAGGGGATAATTCCTTTCCCTGGCACCATCGGGGAGACGCTTTGTCGGCCTCGGCTCCTGGGCGCAGGGACGCCTTAGCCCACGGAGGGTGGAGCCCCCCTCAGACCCGGGCCACCGGCTGGGGTTTTTCTAACGCCCTGCCCCCCGAGCCCCCGGATGGCTCGGGCCCCACGGACTCCGCGCCCTCCAGCCTCAGCTCAGCTCCCCAGGCTTCCCAGACCCAGCGGCGCAGGGGGCGGGGGCAGGGGCAGTGGGGGTTGGAGGGCGCAGCCGGTCCCCAGGGTGGGGAGAGCTGCGGGGGGAGGAGGAGGAGGGTGCCGACGCTTGAGTGGGTTCGAGCCCGAGCCGTAGCCGGGGGAGCCAGTCAGTTTCCGGCCAAGGCAGCAGGTCAGTCCCAGGAAGGGCGGGCGATTGAGCCGAGGGAGCCGGCGGCTGGGCTCTCCTCTCGGCCCGCGATCCCCGGCGCCGCCGCCGCCGCCACCGCCACCGCCACCGCCTTCGCCTTGTCGCCGCCGCCGCTGCAGAGCATCGTAGCTCCGCCGCGCTCCCGCGCCCCGCGCCCCGCGCCGCCAGCCGCCTGGGAGCCCGAGCGCCGAGCCCGGGGCGGAGGAGAGGGGCGCTGGCGCGAGAGCCCGGGCGAGGGAGCCGCGAAGGGAGAAGGGGGCGGGCGGAGGGAGGAGCAGGGAGAGTGGGAGAAGGGGGAGGGAGAGAGGAGAGCGAGGGAGAGCTGGAGAGAGCGAGAGCAAAGAGCGAGCGAGGGAGAGGAGAGAGAGAGAGAGGAGAGAGAAAGACACACGCACGCAGAGACACACGGTCACTGGAATTCCATTAGAAAAAAGTGAGCCGAGCAAGGGTTAGCGGGAGAAGATTTTTTTGAATCTTGTCTTCGTCTTGGTGCGAAAGAAGCGACTCCAGTCTCTCGTCCTCGAAGCTCCGACTGGATTGTTCTTGGGCGCTGACACCC

**GSTP1_CpGLength:800**

GCAAGCTCCGGGATCGCAGCGGTCTTAGGGAATTTCCCCCCGCGATGTCCCGGCGCGCCAGTTCGCTGCGCACACTTCGCTGCGGTCCTCTTCCTGCTGTCTGTTTACTCCCTAGGCCCCGCTGGGGACCTGGGAAAGAGGGAAAGGCTTCCCCGGCCAGCTGCGCGGCGACTCCGGGGACTCCAGGGCGCCCCTCTGCGGCCGACGCCCGGGGTGCAGCGGCCGCCGGGGCTGGGGCCGGCGGGAGTCCGCGGGACCCTCCAGAAGAGCGGCCGGCGCCGTGACTCAGCACTGGGGCGGAGCGGGGCGGGACCACCCTTATAAGGCTCGGAGGCCGCGAGGCCTTCGCTGGAGTTTCGCCGCCGCAGTCTTCGCCACCAGTGAGTACGCGCGGCCCGCGTCCCCGGGGATGGGGCTCAGAGCTCCCAGCATGGGGCCAACCCGCAGCATCAGGCCCGGGCTCCCGGCAGGGCTCCTCGCCCACCTCGAGACCCGGGACGGGGGCCTAGGGGACCCAGGACGTCCCCAGTGCCGTTAGCGGCTTTCAGGGGGCCCGGAGCGCCTCGGGGAGGGATGGGACCCCGGGGGCGGGGAGGGGGGGCAGACTGCGCTCACCGCGCCTTGGCATCCTCCCCCGGGCTCCAGCAAACTTTTCTTTGTTCGCTGCAGTGCCGCCCTACACCGTGGTCTATTTCCCAGTTCGAGGTAGGAGCATGTGTCTGGCAGGGAAGGGAGGCAGGGGCTGGGGCTGCAGCCCACAGCCCCTCGCCCACCCGGAGAGATCCGAACCCCCTTATCCC

**HEMK1_CpGLength:800**

CCGCGACCTTCGAGAACCCGCATGCTGTTCTCCACCAGGTCTCTCAGTCCTCCCTGCCCCAATCCCCATGCCCGCCTCCGCGACCCTGTGATGCCTCCCTTCTTGCACAGGAGCAGTGACCTCAGCACTTACTTAATCCTCTCCCGGCGCCGAGCTCAGTTGGAGAGGCTAGGGGTGGTAGTGACTGGCAGGAGGCCGGGGCGGGGGGAACCCCCAAGCCCGGCGTCTGGGGCTGCGGGTCCGACCCGAGATCCGCCCTCCCTGCAAGCCCCGAGCCGCTGGCCAGGCCCGCTACTGCGCACCAGCCGCATCCGCGAGCGCTGGCTCTGCCGGCCTGAGCTAGGGTGGGTAGGGCCGGGACCCACGGCGGAGGTGGGGCCGGGCCGAGCAGCCTCGGGGGATCCCCGAAGCTACAGCGCCTTGCCTCCCTGCACGCTCCGCGCCCCCGGCCTCCGATTGGCTGTCGGGCCTAGAGCCCGCCCAGAATTGGACCGTTCGCTTGTCGCTCGGGTCTGGCTCCACCCCCAGAGGGAGCCTAGAACCTGGTCGCAGTTTTTAGAGACTACCCTCACCCCGTGGCCTGCGCCGAAGTTGGGCGGAGGACAGTGGGTGGCCAGGCCCTTCCGGGCCAGAACTCGGGACCCCTGCCAGCTACCCGTGCCAGGACAGACTCAAGCCCCCAAAACGCGGATGGATGTACAGAGGAGACTTGGGGAGAGCACTGGACTGGGAGTCCTTGGGCCTGCACTGAACTCTGGCTGACTTTGTGACCTTGAAGAAACTGCTTTTCCCTTCCTGAA

**HOXA7_CpGLength:550**

CAGCAAGACGCAAAGAGAAAAGACCAAGGCCCCCGCCGCCGCCGTCTGTCTAGACTCAAGCGACTGAAGGGGCCAACAGAGCTGGTGTTTAAAGTAGAACCTGCCCAGTCCAACAGCCCGAGCAGGGAGCGATTTCGGGGATCGCGGGAAGGAACGCACTTCGCCAAGGGAGGGCCGGGTGCCCTCGCCACCGGCTCATTCCTGCTCCGGTTTTGCCCGATGCGCGTCCAGGAGGTTCTGGCAGGACGCACTGCCCCTCTGCCCCGGCCAAGGAGGATGCGGATACTGCCCGCAAGGCTTCGGCCTTTATGGACCCAAGTCAGCCAACTGGGCCGAGTCCTGCGGACACCGAAACCTCCCTTTCGTTTCCAGGCTTCCTTCTCCCCTCTTGCCCTCTGTGGTCTGATTTAAAACGAAAAGGTCGGATAAAATCAGGCTTTCAATAAGGCTTCTTTAACTGTGTGTTCTCTATTCATTGGTTCTCTACTTATTTGACTGAAAAGACACAAATGCACTAGGTTATGTGAGATAATTTTCACAGAAATACTCA

**HOXB5_CpGLength:550**

GCTTCCTCATCCAGGGGAATATTTGCGGAGTCTGCCCCTCGGGCGCGGCTGTGGAGGTGGCCATGGGCTCTGGCTGCGCCCGAGCTAGGCTGGGGCTGCTTAGCTGGCTTGCCGCTTCCTCAGGCTCCGAGGACGCGCTGGCCTCGTCTATTTCGGTGAAATTGGCGCTGGAGCTGGCTGAGGTCGCCTGGTCGGAGGGGGACGAAGCAGAGGGCTTGGCGCCGTGGCTGTCGCCGTTGGTGCAGGGCAGGGACTCGGGCGAGGACAGGGAGCAGCTCGAAGCCGCTTGCCTGAAGCGGGGCTCCTGGGCGGGCGCGGGGAAGGCGCGCGAGCTCTCGCCCACCGCCCCAAAGTGGCTGGAGGAGGCCGAGGAGCGGTTGACGCTGAGGTCCATCCCATTGTAATTGTAGCCGTAAGAGCCGGTGTGCATGGCAGCGGGATCCCTGTAAGAGCCGCTCAGAGAGCTGCCACTGCCATAATTTAGCAACTGATAGTCCGGGCCATTTGGATAACGCCCCGAGAAGGAGTTTACAAAGTACGAGCTCATTTG

**HOXD3a_CpGLength:1300** TGAAGAAGGGGCCAAGACAGGAGTCCCAAAATAGATGGGGGTGCTTGTTCCAGACAGGAAATGAGCAGAATGCATAAAATCAGACATCTGTCCTTAATATATGGAAGGGCTTGCCCCAGACGCGTGGACATGTGTATTTATAAGTGCGCAGGCAGAAGTTTAAATATTCAGACACATTTTATGATTGTCCTCTGCCCGTCTTCTGCTTAATGGCTCCTCCCGTGCCCACCAATACATCAATACCGTTGTGTGTATTGAATCGCGGGCAGGGATGAGGAGGAAGCGAGTCCATAGCAGAGCCCGCTGAGTATAGAGTACAGTAAATCGGGACCCTCGGCGGACGGCGCTTCCCGCCCGCCTGCCCGCCATGTTGGGGAGCCCTCCCTGCCCCCCGCGCCGGGCTGGGCGGCCGGGGCTCGCTGGCAGCCGGGGGAGGGCCTTTCATAACCCGGAGAATTTTCTAAGTGCGAGGAAGATGATAAGAATAGATTTCTACAAGTCCCGACCACGTGATTGGCGAAATAATTAATTCAGCACGTCCCTTAAGAAACACGGAGTCGTCATTAATCTGCCACGCAAAGGGCTCTCTCCGACTTGGAAAGTGCAGGGATCCCAAGAATATCACCCGTCCAGGGGGGCCGCGCGGTGCCCCCGGCCCTCCACCCCCGGCCCCCGGCGGGCGCGGGAGCGCGGCCGCAGGTAAATATTTTGGCAACTTTTATTTCATCAGATTTAAATCCTTAATGAACTTAGCTGTCACGGCCGCTGACAAATAGTTCCCTTTGCTTCCTGATTTGGAACTGCGCGCCGGCGAGAAGTTGTTAGTGGCTTGGATGGTGACCTTTGGTTCAGCAAAGCTTTGCACTTATGAAAAATTACTGAGAGCGGCCGAGTGTGTGTGTGTGTGCGTGCGTCCGCGCGCGCGCTTGTGTGTGTGAGAGAGGGAGAGACAGAGACAGAGACAGAGATAGGGAGAGGGTGTGTGTGCGAGGCGCTAGGGTGCCAGGGGGCGAGGGGTGAGGGGCGAGGTGCACGCGGGCTGCTCCAAGACAACAGGAGTTGTAAAAAACCGGCCCGGCTGGCGGACGCGCCTGGCGCACGCGGGCCGAGGTTGCCTGGTCGCCTGTGTCTACCAGGAACAATGGTCGCTGTCACGGCATCTGCCGCCTATTCTTAAACCGGTGAGAAAAGGCCCTGGCCCTCTTTTCAAGCGAGGGTCGTAAATTTTTCTTTGCGTCATAATAGAAGGCTATAAAATCGAGTTGAAATTTTACCCCAGGCAGGTTTTAACCAACAGATA

**HOXD3b_CpGLength:800**

TCTCTTTCAACTTGGATGGGCTGACTCTACCCGTCGGTGATTTACGACGATTGCAGCGCTAGTCACAGCCTGGCGCCTGGTGTCCCCTCCCTTCCCAAGCCCCCTCAGCTTTTCCACTGCCACCGGCGTACAAGCAAGTGCCGAGCCGGCCTCCGCAAGTCGGACTAGCCTCCCGGCGTCCGAGGCCACCACGGGCAGCAGATTTTTGGTCCCCAGCGAGGCTGCGCGCGTTCGTCCCGCCTCCGACCGCCGAGCAGAGCTGCTAGCAGAAGCAGGCGCCGGTCACTTTATATAATCCTGCTGCTCGCAGGGTGCAAGAGCGGGAAAAGTGCGGAGTAGGGAATTCTTTTGCTGCGCTGCCTCCTACGCGGAGCCTGCTTTCCACTGCTGAAAAGTGCCGGGCCTTGGGAAGTGTTTTTCTTTTCATTCCTTACCGAAGCGTTTACTGCCGCCGTGGTCGCAGTCATAAATTTTGCTACAAACCACAATGACAGGTGCATTGATATGCACCGTGAGAGCTCCAGCTGCTTAATAACCCCGTCCCCTGGTCGCTGTGAGCGCCTTTTATTTATTTGGTATCATATTAGGTATTGATCTCTAGTAGCATTAAGTGCGGTGAGCAAGTATCAGGGTTCGGCTGCTTTGGAGGCGCAGCGGTTGCGGCGGGCCGGCGGGCCCGGGGAAGCGGGCGGTGGCCGCTCAGAGAATACCTTCCTTCCGGCAGGAGACCGTTTGGCCCTGTATTCCGGGCCTGCGGTTGGGCCTCCAAGCTGAGTTGGGCAACTTCCCAGCACCGCAAG

**HOXD9_CpGLength:2050**

CGCGAAGCGGCCCTGCCCGGGGAGACTCGCTGAGGCAGGGCTGAGGCGGCGGGCGGGAGCAAGCTGCTCTAGCATTTGGGTTCTGCCCTGTGGCGTGTTCTCTTCCAGGGCCTTTCCAGCATCATCGGAGAAGACGAAGCACCCTGGCCGCCACTGTCCGTGCTGCGCCAACTCGCCCGGCCGCCCGCCCTTCCGAGGGCAGGCAGAAGCCCCTCTGTGTCCTCCACCGCCGCGCCCCGGCTCGCCCCTCGGGCCGCGGCGTGTGCCCAGCCTCACGTCGGGGTGTGTGTGGCCGCGCGGGCGTGTGTGAGTGTGGCAGGGGGAGGGGGCCCTCCGATCTGCTCCATCCGTCCGTTTTATTAGGGACACATTAATCTATAATCAAATACACCTCATAAAATTTTTATTGAAAGGCATAATATCATTACAGAGGTCTTCCACCTGTTTTAAACAACACGACAAGCTGTGAGCAAGCGTGTGTGTGGGGATGTGTGGGGAGGGGTGGGTGTGAGTAGGGAGAGAGGCGAGGGGAGAACAGCTCCCCTCGGGCGCTAGGGGCCGCCCCGAGGGCCCGCCTGCCTCGGGCGACACCGGCCTGGCGCCCCCGCGGCCGCTCCGTGTGCCCTGGACTCGCCGCCCGCGGCTCGGAAGCTGGAGAGTCAGCGACGGGGCCCGACTGCGGGACCGAGGGCTGCAAGAAGAAGCGAACAAATAGTCCCCAGCGCCTCCTCTGGATGCGGTCGCGTCTGTGGTCCTGGCAGCCGCTGGGCGGGCCAGGCCAGGTCGGGCCGGGCCGAGCCGGGCACATGGACCTGGGCCTGCGGGCTCTAATTGCGGCGCTTATGTTGATGATTTTTTTTTTAATCACAGCAGCCCCCAGTTTAGCGGACTGATTTACTCCCGGTATTGGTAAATATGATCACGTGGGCCGCGCGACCAATGGTGGAGGCTGCAGCCTGCGAACTAGTCGGTGGCTCGGGCGCCGGCGGGGAGCTGCTCGGCGGCGGACAGTGTAATGTTGGGTGGGAGTGCGGGACGCCTCAAAATGTCTTCCAGTGGCACCCTCAGCAACTACTACGTGGACTCGCTTATAGGCCATGAGGGCGACGAGGTGTTCGCGGCGCGCTTCGGGCCGCCGGGGCCAGGCGCGCAGGGCCGGCCTGCAGGTGTGGCTGATGGCCCGGCCGCCACCGCCGCCGAGTTCGCCTCGTGTAGTTTTGCCCCCAGATCGGCCGTGTTCTCTGCCTCGTGGTCCGCGGTGCCCTCCCAGCCCCCGGCAGCGGCGGCGATGAGCGGCCTCTACCACCCGTACGTTCCCCCGCCGCCCCTGGCCGCCTCTGCCTCCGAGCCCGGCCGCTACGTGCGCTCCTGGATGGAGCCGCTGCCCGGCTTCCCGGGCGGTGCGGGCGGTGGCGGTGGTGGTGGAGGCGGCGGTCCGGGCCGCGGTCCCAGCCCTGGCCCCAGCGGCCCAGCCAACGGGCGCCACTACGGGATTAAGCCTGAAACCCGAGCGGCCCCGGCCCCCGCCACGGCCGCCTCCACCACCTCCTCCTCCTCCACTTCCTTATCCTCCTCCTCCAAACGGACTGAGTGCTCCGTGGCCCGGGAGTCCCAGGGGAGCAGCGGCCCCGAGTTCTCGTGCAACTCGTTCCTGCAGGAGAAGGCGGCAGCGGCGACGGGGGGAACCGGGCCTGGGGCAGGGATCGGGGCCGCGACTGGGACGGGCGGCTCGTCGGAGCCCTCAGCTTGCAGCGACCACCCGATCCCAGGCTGTTCGCTGAAGGAGGAGGAGAAGCAGCATTCGCAGCCGCAGCAGCAGCAACTTGACCCAAGTAAGTGCAAAAGAAATTGCCCCCTGATTTATTGCTGAAACCTGTAAGGCTCGAATGTGCAAAACTGATAGTTTTACTAACCTATAAAAACGTCTAGACGCCTACCCAAGCCTAGGCGAACAACATGCATCCATAAAAAGAGCTTCCCATAACCACCTACCCTGGGCGCTCAGTTAGTACGGTAAACAGAGCGCGAGCATTAAGGCTTTTTATGATAA

**HOXD10_CpGLength:550** GGGATGTCTCAGGGCCCGCGGAATCCGACTCACCTTCCCGCTGGGCTGCCCGGGACTAAATTAACCAGCCTGCGCCCCCACCCGCTTGTCCTGGACCCGGCCCTCTTAAGCGCGTTCTGCCTGGTTGTGTGTGGGGGGAATGCTTCTGTGCGCTGGCGCCAGGGCACTCTGGCTTCCCTCCCCGTCCGTGCGTGTCCACTTGGAGGCCCCTAGAGCTGAGACTTTCCTTCCGCCGGTTGGGCCCAGGGGCCGAAGCGGGGGACGCGAGTGGGGCGGGCTGGCCGAGCGAGCCCTGGAGAGGCGGACAGGAGGGCGGCGGAGAGCGCTGGGCCGGTTGTCTCCAGCGCGCACTATCGCGGGCGCGTAGTAGATGTCGCTGTTGTCCGTGCTTACCCGGCCGGCCGGCCAGGCTCTGGAGCACGTGACCCGAGAGGAGGCTGCGGCTCAAGGCCATTTTCAAATCTCATTGGCTTGGTTGTCATGTGGTCGGCAGAGGCATCCACAATTACACGGGGAATGTTTTCCTAGAGATGTCAGCCTACAAAGGACA

**KIFC2_CpGLength:1300**

TTCACTGGCTGCGGAGGTGGAGGGGTGACTCTGGGAGCTCAGGGGCAATGGATCCAAAGA

GGCTAGGGAGCTTGTGCCCTGGGAGACGGGGTACAGGGACTTTACTACCGCCTCTGCCAG

CAGAGACCTGGCCTGAGACCCCATGGGCAGGGCCGCGACCCAGGACTAGAGGGGCAGGCC

CTCTGCGGGCGCGAGAGCCGAGCCCGAGCCGTTGTCGGGACTGGGGCATGGAGGACTGCC

AGGGGACGGCGTAGGGGTGCAGGGGGTCCCGGTGAGCGGAGTGTCGGTGCTGAGGGAAGA

AGGCGTCCCGGAGGAGCGCGGGACCCTGCGGCGCCGGGCTGGCCCCAGCTCCACTTGACC

CACTCGGTCGGCGAACTTGAGGGAGCAGACTGTCTCCCCGAGATCCTCCGGCCGCGTGGA

GATCTGCGGGCAAGAGGCGCGGGTGGGCGGGCCCGGGTGGGCGGCGACCGGCACGCACAC

ACCTGCCCCGCCCCGGCGCCCACCTGCAGCAGCAGCACCGCGGTGGTGCCTGGGCCCAGC

GCCGGCTGCAGCAGTCGCGTGAGCTGCGAGTCGCGGAAGGGCACGTGCGGCCGGTGGGCC

CGCAGTGCGGCCATCACGCCTCCTAGCGCCAGCAGCGAGCGGTTTATGGTCTGGGCCTCC

CGCAGGCGCCGGGCGCCGTCTGGGTCTCCCCGCGGCGGGCCGGCCGCCCCTGCCTTCCGT

GCGCGTTCGGATCCCGCCAGGTCCACCAGGTGCAGCGTGCCTAGAGGGGCGAGCGAGCCC

GACATGGGGGAAGGCCGGGCCTCGGGTGCTCTGGAGACTCCGCAGGGCTCAGGCACCGGC

CGTGGTACCTGCGGTGCCTGGAGCGCGCGGTGGAGACGCCGCGCGCAGCGTCAGCGTGAC

CAGGGCATGCGAGCGGGAGCTGCGCTGGTTCATGGCGGTGGCGGCGGTGGCCCGGTTGCT

CCTCCCCAGTTTCAGCATCTGGAAGGCGGGGCGAAGGGCTGGTCAGGTGCAGGGTCCCCG

CCCCCAAGTGGAAGCACGGGGCGGGGTCTCGGAGAGCGGTGCAGCCCTACCTGGTGCAAT

GTCTCCAGGTTGGGCACGTCCCAGTGGGTGAGGCCAGCCACCTGGATCCCGCCCTGGCCT

TCTGGGCCCTGCCTCACGGCCAGGCGCTCGGGAGGCCCTGGAGCAAGGAGGTCCCTGGTG

AGAAAGGGGAAGGTCTTGGTTCCAGAATTCTCTGGAAATCCGGAAGGGCCCTGGGCCAGG

GGTGGGGAGAAGGCCTCCCTGGTGGAGTAGCCCACCTGAC

**MOXD1_CpGLength:1050**

CTTCTCCCTTCCCATCAGGGATGAAGCGGGGTGTATTGCAGGTGTGTAGGCGGGGGGGCGTCTATTAAGTGGGGCGCTGGGGACAGGAAAGTGTTCAAAGAAGGAAACCTGCCTGGGGTCATCCGTGCTCGGCTTCCCTACCGCGCGCCTTCTGGGGCTCCGGGAGAGCAGGCGCTGCCCGCGGAGGCGAGAGTGAGCGTGGCCGGGGGCGCGGGGCTGCGCCAGGTGAGAGAGAGCTGCGGGCCCCGGGGACGACCCGCACCTGCGCCCTCTCTGGGGTCCGGACGGGACCGGGCTCGGCCGGGCGGGCTCCGGGAGGAGACGCGCTTACCTGGAGGTAGGGCCGCCCGTGGGCCACCCCGCCCACGACGATGTCGGCGGACGCCATGGCCCCGGTGGGCGAGAAGCCGAAGCCCACGTAGCCTGCAGTGCGCACCTGGAGGCGGAAGGCGATCTGGCTGCCCCGCTGGCTCCAGCCCAGCCAGTACTTGCCCTCCGAGTCCAGGAGGGTCCGGTGCGGATAGGTTCGGCCCGAGCCCCCCGCCGCCGTCCCGGGGAGCAGCCCCCACAGCAGGAGCAGCGGCCAGCAGCACATCCTCGGGCGCCTCCTGCCCGCCGGTACCGGCCTCCAGCCGCTGGGGAGTGAGGAGCAGAACGAGGAGCGGCAGCGGCGGCGAGCGGGAGCCCAGGGACCGCCCCTCGGGCCGGCCGCGCGCCCGCCCGGGAGGGGCAGGACCCGCTCTCCCCGCCCCGGGCCGCCTGCAGGGCGCGCCGCGTCCCGTCCGGTCCCGTCCCGTTCCGTCCCGTCGGGTCCCAGCCCCGCCAACTCTCACCCTGCCCGGCCCTTCCAGCGGATCCACTTGCAGCCCCCGACCCCTGACTTGGTCCCCGAGGTCGGGGCTCGGTGGGAAGGAAAGCGAGGGGCTTCCCGGAAAGAAAGGGAGGAGGAGGCAGGAGGAGGCAGACGCCGAGAGAGGAGAAAAGGAGGCCAGAGGCAGAGGGAAAAGGAGGAGGGCAAGAGAATCCTAGAGCGGGAAGAGCGTGGGCTCT

**NEUROG3_CpGLength:1300**

GTGGCCTGACCAGAGCCACACGAGGCTCTTCTCACTGGGCGAGGCTCTTTGAGGAACCGAGAGTTGCTGGGACCCAGCCCGCCCTCGAGAGAGCAAACAGAGCGGCGCTCCCCTCCCCCGACCCCGGCCCTTTGTCCGGAATCCAGCTGTGCCCTGCGGGGGAGGAGCGGGCTCGCGTGGCGCGGCCCCAGGGCCCCGGCGCTGATTGGCCGGTGGCGCGGGCAGCAGCCGGGCAGGCACGCTCCTGGCCCGGGCGAAGCAGATAAAGCGTGCCAAGGGGCACACGACTTGCTGCTCAGGAAATCCCTGCGGTCTCACCGCCGCGCCTCGAGAGAGAGCGTGACAGAGGCCTCGGACCCCATTCTCTCTTCTTTTCTCCTTTGGGGCTGGGGCAACTCCCAGGCGGGGGCGCCTGCAGCTCAGCTGAACTTGGCGACCAGAAGCCCGCTGAGCTCCCCACGGCCCTCGCTGCTCATCGCTCTCTATTCTTTTGCGCCGGTAGAAAGGTAATATTTGGAGGCCTCCGAGGGACGGGCAGGGGAAAGAGGGATCCTCTGACCCAGCGGGGGCTGGGAGGATGGCTGTTTTTGTTTTTTCCCACCTAGCCTCGGAATCGCGGACTGCGCCCAGTGACGGACTCAAACTTACCCTTCCCTCTGACCCCGCCGTAGGATGACGCCTCAACCCTCGGGTGCGCCCACTGTCCAAGTGACCCGTGAGACGGAGCGGTCCTTCCCCAGAGCCTCGGAAGACGAAGTGACCTGCCCCACGTCCGCCCCGCCCAGCCCCACTCGCACACGGGGGAACTGCGCAGAGGCGGAAGAGGGAGGCTGCCGAGGGGCCCCGAGGAAGCTCCGGGCACGGCGCGGGGGACGCAGCCGGCCTAAGAGCGAGTTGGCACTGAGCAAGCAGCGACGGAGTCGGCGAAAGAAGGCCAACGACCGCGAGCGCAATCGAATGCACAACCTCAACTCGGCACTGGACGCCCTGCGCGGTGTCCTGCCCACCTTCCCAGACGACGCGAAGCTCACCAAGATCGAGACGCTGCGCTTCGCCCACAACTACATCTGGGCGCTGACTCAAACGCTGCGCATAGCGGACCACAGCTTGTACGCGCTGGAGCCGCCGGCGCCGCACTGCGGGGAGCTGGGCAGCCCAGGCGGTTCCCCCGGGGACTGGGGGTCCCTCTACTCCCCAGTCTCCCAGGCTGGCAGCCTGAGTCCCGCCGCGTCGCTGGAGGAGCGACCCGGGCTGCTGGGGGCCACCTTTTCCGCCTGCTTGAGCCCAGGCAGTCTGGCTT

**NODAL_CpGLength:1550**

TAAAGCTTCCCCAGAGGGAGGAAAGGTGGGGGCGGGGCGGCTGCTGAGGCCCAGGATATAAGGGCTGGAGGTGCTGCTTTCAGGCCTGGCCAGCCCACCATGCACGCCCACTGCCTGCCCTTCCTTCTGCACGCCTGGTGGGCCCTACTCCAGGCGGGTGCTGCGACGGTGGCCACTGCGCTCCTGCGTACGCGGGGGCAGCCCTCGTCGCCATCCCCTCTGGCGTACATGCTGAGCCTCTACCGCGACCCGCTGCCGAGGGCAGACATCATCCGCAGCCTACAGGCAGAAGGTAGGCAGTGCCGCGTGCCGCGCCCTGCTGGGCACCCCCGGGGCGCCTCCGCCGCGTCCAGCCAGCGGACTCGGGAAGTGCTGTGGGTTGGGGGCTGCGGCTCCGAGCCGGGTTTGCAGCCGCCCGGGCGTCCCGAGCCCAGGGCCTAGCTCTGCGGGTGTCTCCGCGTCAGCAGGCTCGGGGTGCAGCGTTGGTGGCTGGGGGCGTATCCACGGCCGAGTCGGGAAGGGATTCTAGCGTTCAGGGTGTGTCCTCGACGGGGACCATTGTCTCTGGGTTTTGGTTTGGGATTGCGCGGAGCGCAGCGCGGAAGGGTGGGAGCTTCTAATCTCCAGTCTTGTGAAGTTGCTTATCCCGGAGCCTGGGTCTGCGCATCTGTAGGATAGGTGTAATAAATAACACCTCGCCTATCAGACTGTGGAAAGCGCGAGATGACAATGCGCGCGAAACGCTCAGCGCAGTACCCGGCACAGCCACAGTCAACGGTCGTTGGTATTACTGTAATGGTTTGGTCTTGGCGATTTTTTTTTCTTTCTGCGAGTGAGGGTGAATGGGTCCCGGGGTGTGACGTCGGGAGTATCGGCAGCTGAGCTGGTAACATCGGGGATTCGGGCTCACGGCCCGGAGATCAGGGATGGGCTGTCCCGAAGTCGCGAACTGTGGCAGCCTTGGGTCCTCCAGCCGCGCCGGGGAAGTGTCAAGTGTCTCGCTTAACCCCGGGTTCGGGGCCATGATTTGCAGGGGAGTGGGTGTCAAGGACGGCAGGGATCTGAGGGTATCGCCCTCGAGGACCTGGCAGCGCGTTCTGGGCACCCAGCGCGGCGAGCAGGTGGGTGCTGCGGAGAGGGAGCCCCTTCCGCGCCTCAATCCACATTCTGCCGCCTGGGCAGCCGCGGCCGCCCACGCCTCCCTCCGCCTGCGGGGGCCAGACGGCCCTCCCTGGGGCCGGGGCGCAATCCACAAACGCTAATCTGATCCGACCTGCCGCCTGCCCGCCCCTTGTGACCTGGTGCCGGGGGCCCTTCGCTCCCGCGCCTGGGGTCAGACAGCCGGTGACCCTCTCCGGAAGGGTCATCTGGGGACCAGCCAGACCAGGGGACACCCTCGGGGGCGGGGCAATGAGAAATTTGCTGGAGTGCTCGGCCCCTCAACCGAAAAGCGGCCGGGGATGGGAGGGGGCAAAGAAGGGAGGGAGCGCTTTTCCAGTTCACTCCCTTCTGGAAAGTTCGAGATGTGTGCGGTGATGGACAGGCATCTG

**RASSF5_CpGLength:1550**

AGGGGAACTGGTATCTCCACAGTAATTACTAGAGCAGCTCTGGGGAACGGAGGGTTGGCTAAGGAAGAAAAGCTCCCCCAACCCTTGGGGCGAGGGAGCGTTCTCTCAATGGAGCCCCCCCAACTCCCCTCCACCCCCCACCAGTCTTCCAGGAAAGAGGAATACCCTACCCGGCAGGGCTGCGAAGGAAGGGGAAATCCAACCAGAGCGAAAGTCGCACGCGGACAGCTCTGCCAGCCCTTGGAGGCATCCGGCGGTCACCCACGGGACAAAGCGCGGCTGCGGGAGCGCGCGCGGGGCATTCCGGACCCGCGTCGAGCTCCGCTCTAGAGGGGGCGGCGGGCGGCGACAAGCCGGAGAGAGGAAGGGCCAAGGAGCACGGCCCTCCTGTCGGCACCATCAGCGGGAGAGTGGCGAGCGGACGCCTAGACGGAGGGGCCCTACTCAGACCCCATCGAGCCAGTTCCCAAGCTTTTCCCTCCGACCTGCTCCCTCCCGGGGCGCGTGAGGGTGCGGGTCGGGGGTGAACCTGGTGTTGGGGAAAGTGATTGGCAAGGGAAAGAGGCAGGGCTGAAGGCCTAGGGCCCCCCGCAGAAATGCGCCCTCTGAGCTGTACTGTACAGAGGCGCTCCTCTGCGGAATCCTGCAGCTGCTCCAGGTGAAGACGCCCCCAAATCCACACTCGCGCAGACGTCGCCTGGCACGGACCCTACCCCCTTCGGTCCGTCGGCGGTTCTCTTGGGTCGTCCTTCCTGCCACTCCGACTCTCCCCGCCCCCGCCCCGCCCAGCAGGCTGCGGTTCCTTTAAAGGCGCGCTGGTGTGGGGCGGCCCCTTCTCTCGGGGCTGGCTCGGGAGTAGCGCAGTCGCCAAAGCCGCCGCTGCCAAAGCTGCCGCCACTAGCCGGGCATGGCCATGGCGTCCCCGGCCATCGGGCAGCGCCCGTACCCGCTACTATTGGACCCCGAGCCGCCGCGCTATCTACAGAGCCTGAGCGGCCCCGAGCTACCGCCGCCGCCCCCCGACCGGTCCTCGCGCCTCTGTGTCCCGGCGCCCCTCTCCACTGCGCCCGGGGCGCGCGAGGGGCGCAGCGCCCGGAGGGCTGCCCGGGGGAACCTGGAGCCCCCGCCCCGGGCCTCCCGACCCGCTCGCCCGCTCCGGCCTGGTCTGCAGCAGAGACTGCGGCGGCGGCCTGGAGCGCCCCGACCCCGCGACGTGCGGAGCATCTTCGAGCAGCCGCAGGATCCCAGAGTCCCGGCGGAGCGAGGCGAGGGGCACTGCTTCGCCGAGTTGGTGCTGCCGGGCGGCCCCGGCTGGTGTGACCTGTGCGGACGAGAGGTGCTGCGGCAGGCGCTGCGCTGCACTAGTAAGTGTGAAGGCAGGGGAGGGGCGTGCGGGGAGACCGCAGTCTGGGGGCGAAGGACTGGGAGGGCCCTGGGGCAGGGAGAGAGGGGCCATTACACTCTTTGGCTCCAGGGGAGCCCCGAACATAAGGAGATAACAACCCCTGGGGACAGTCCGCTCCTTAGTTCCCTGCCCGGTATCCTTTAGT

**NSD1_CpGLength:1100** CTAATCACAGAAGGCTTTCTGGAGGAGGCGGAATTTTTATGGCGGCCGGATCCGGCTTTCTCTGAACAGCGAGAAGGCGCTTAGCGCCCTAGGGACCAGGTAACTCCTGAGGTGAGCTTCTTGGTGGGGATCAAGCCCAGGGGGCGACGGAGTCCGGGCTGGGGGAAGGGCCCGAGGGGCTGGAGTCGCAAGTTCAGGCCCAGCTTGGGCTCCCTGTCCCGCCCTTCCGCTGTCTTGGGGGATTGGACGCCACGCGGTCGTGCTAGATTCGGTGCTGCGGGCCCGGTGCAGGATGCAGGCCGTGAGGCCCCAGGCCGAGGGCTGCGCCAGCGGGCTTGTCCCGGCCAGCCGGGCGGTCCCGTGTCCCGGCGCAGCTCCGCTGGGGTCCAGATGCCCGGCCCTCAGGGGCGAGGCGCGCACTCCCCGGGGAACCGGGCTGCGGAGCAGGCGGCCCGCTCTGGGCGGCGGTGGCACGAGAGGGCCATCTGCCTGGGTGCCGAGAACTGCAGCGTCCGCGGTGCGAGGCGCGGCCCGTCCCGTCCCGGCCCCCAGCCCGGCGCGCACGCACATACCCACGCCGGCCGGCGCCCGCTGCCCGAGCCCCCGTGCCAGGCCCAGACCTTGACTAGGCGCGGGAGGCGGTGCAGGGACTAGAGGACCCCCTCCCCCGGCGTTCCCCTCGCCCCGCCCGAGGCTGCGAGGACCCCTGGGCTCGGGGGTGGTGAGGGAGCTTCGTCCCGGCTGGGCCCGGGCTGGGGACTCGGCCTCCCTGGGCGGGGGCCGCACGGCTGCAGGCCGAGGTGCGGACGCGCTGTCAGGCTGCAGCCCGGCTCGGTGCCGGGGGTGGGCTCAGCGCTGGGGTCGCCTGGCTTCGTTCCCCCGCGGAGGCCACGGCCGGGCGAGCAGTGCCGGGGCGGGTAACCCGACCCGGCTCCCCAGAGCCGCTCACCCCGCACGGCCCGGCAAGGGGAGGGAGAGGGATGGGGGGAGGGGGAAGGGAAGGGGTGGTGGGTGAGGGGCTGTGGGCACCGCAGGGCCGAGTCCCCGGCCCGTCTGCGCTGCTGTAGGGCGGCTGCCCGCGGCACCCGGGACGATCCAGC
