# Supplementary material for: A panel of DNA methylation markers for the detection of prostate cancer from FV and DRE urine DNA
Source: Clin Epigenetics. 2018 Jul 3;10:91. doi: 10.1186/s13148-018-0524-x (PMC6029393; doi:10.1186/s13148-018-0524-x)
Supplement: Supplementary file 3 — Table S3. List of CpG islands included in the biomarker panel. Table S4. List of primers and probes. (DOCX 41 kb) [file 13148_2018_524_MOESM3_ESM.docx]

**Additional file 3: Table S3. List of CpG islands included in the biomarker panel.** Table S3 shows the list of the CpG islands and associated genes and chromosomes. The coordinates of the CpG islands on the chromosome are listed for build 38 (annotation release 108). The position relative to the first untranslated region (UTR) is ”–“ if it is 5’ to the UTR or “+” if it extends into the UTR based on the mRNA ID provided.

| **Gene Symbol** | **Gene ID** | **CpG Length** | **Chr** | **Chr Begin** | **Chr End** | **mRNA Id** | **Pos relative to 1st UTR** |
| --- | --- | --- | --- | --- | --- | --- | --- |
| ADCY4 | 196883 | 1050 | 14 | 24334354 | 24335403 | NM_139247.3 | -332 |
| AOX1 | 316 | 800 | 2 | 200585665 | 200586464 | NM_001159.3 | -343 |
| APC | 324 | 550 | 5 | 112737440 | 112737989 | NM_000038.5 | -419 |
| CXCL14 | 9547 | 850 | 5 | 135578447 | 135579496 | NM_004887.4 | -217 |
| EPHX3 | 79852 | 2340 | 19 | 15231708 | 15234047 | NM_024794.2 | -1627 |
| GFRA2 | 2675 | 1800 | 8 | 21788634 | 21790433 | NM_001495.4 | -1599 |
| GSTP1 | 2950 | 800 | 11 | 67583465 | 67584264 | NM_000852.3 | -130 |
| HEMK1 | 51409 | 800 | 3 | 50567331 | 50568130 | NM_016173.4 | -1821 |
| HOXA7 | 3204 | 550 | 7 | 27158531 | 27159080 | NM_006896.3 | -2403 |
| HOXB5 | 3215 | 550 | 17 | 48593136 | 48593685 | NM_002147.3 | +56 |
| HOXD3a | 3232 | 1300 | 2 | 176156754 | 176158053 | XM_005246513.4 | -409 |
| HOXD3b | 3232 | 800 | 2 | 176164754 | 176165553 | NM_006898.4 | 677 |
| HOXD9 | 3235 | 2050 | 2 | 176121754 | 176123803 | NM_014213.3 | -931 |
| HOXD10 | 3236 | 550 | 2 | 176116254 | 176116804 | NM_002148.3 | -510 |
| KIFC2 | 90990 | 1300 | 8 | 144472255 | 144473554 | NM_145754.3 | 1688 |
| MOXD1 | 26002 | 1050 | 6 | 132400832 | 132401881 | NM_015529.3 | -347 |
| NEUROG3 | 50674 | 1300 | 10 | 69572416 | 69573715 | NM_020999.3 | -261 |
| NODAL | 4838 | 1550 | 10 | 70440217 | 70441766 | NM_018055.4 | -57 |
| RASSF5 | 83593 | 1550 | 1 | 206506696 | 206508245 | NM_182663.3 | -834 |
| NSD1 | 64324 | 1100 | 5 | 177131824 | 177132923 | NM_172349.2 | -1255 |

**Additional file 3: Table S4.** **List of primers and probes.** Table S4 shows the list of primers and probes used to assay CpG islands methylation. The Mix number refers to the multiplex used for the primary amplification. Primers BNF and BNR were used for the primary multiplex amplification as described in the manuscript. Primers F, R and the probe were used for the qPCR amplification. CF and CR are the primers used for the control amplifications to verify the recovery of DNA following bisulfite conversion and multiplex amplification. Markers in Mix 1 were analyzed using DNA deaminated at 80^o^C for 42 min bisulfite. The rest of the markers were analyzed using DNA deaminated at 70^o^C for 14 min.

| **Assay** | **Primers and Probes** | **Mix** |
| --- | --- | --- |
| ADCY4 | BNF: TAGGAGGGTAGGATTTGGGGTTG BNR: CTATCRCCCRAAACTCTAAACCAA | 1 |
|  | F: GTAGCGGAGTGGGTTAGGTTC R: CCGAAACTCTAAACCAAAACGCAA |  |
|  | Probe: TTTGATGGCGGAGTTACGTTCGTCG |  |
|  |  |  |
| AOX1rc | BNF: GTAGGTTTGGGGGTAGAGGAAG BNR: AATCCCAAATACCCRCTACTTCC | 6 |
|  | F: TACGTAGAAGAGTAGYTCGG R: CTCCGCCTCCCGCTCCGA |  |
|  | Probe: TCGCGTTGGTTCGAGGGYTCG |  |
|  |  |  |
| APC | BNF: GYGGAGAGAGAAGTAGYTGTGTAA BNR: ACAACACCTCCATTCTATCTCCAA | 4 |
|  | F: TCGTTGGATGCGGATTAGG R: CCTAACGAACTACACCAATACAAC |  |
|  | Probe: CGTCGGGAGTTCGTCGATTG |  |
|  |  |  |
| CXCL14 | BNF: GAGAYGGYGATAAGGGGAG BNR: CTCCCCATATCCCTACTCCCAC | 4 |
|  | F: TTATCGTTTATACGCGCGGT R: GCCGCGCCCCTCCGATCAAC |  |
|  | Probe: TAGCGCGGYCGTTAGGAGT |  |
|  |  |  |
| CXCL14rc | BNF: GGAGGGAGGTTGTGGAGAGAG BNR: AAACRCCACCCARCTCTACTC | 6 |
|  | F: AGCGTAGCGTACGGYTATAG R: CCGAAACGCGCCTTCCGAC |  |
|  | Probe: CGACGGCGTAGTCGGAGTTAG |  |
|  |  |  |
| EPHX3 | BNF: GTYGTTGTTGTAGGTGTTTTTGG BNR: CCACCRAAAACACCAAACTCCACA | 5 |
|  | F: TCGGAGCTGGTGGTGATC R: AAAACGCGCAACARCTTCAAC |  |
|  | Probe: CGTCGTCGCGTTTGTCGTTGAAG |  |
|  |  |  |
| GFRA2 | BNF: GAGTTTAGGGAGTYGGGAGYTGT BNR: ACAAACCTACCTAAACCRCCC | 5 |
|  | F: GGACGAGYTTTTCGGAGTTT R: AAACTCGCACCCCACCCCGA |  |
|  | Probe: AGTCGGTTGCGGAGTTTCGG |  |
|  |  |  |
| GSTP1 | BNF: GGATTTGGGAAAGAGGGAAAGGTTT BNR: CTCCRCCCCAATACTAAATCAC BNR1b: CTCCRCCCCAATACTAAATCAAC | 4 |
|  | F: TAGYTGCGCGGCGATTTCGG R: GACGCCGRCCGCTCTTC |  |
|  | Probe: AAATCCCGCGAAMTCCCGCCGRC |  |
|  |  |  |
| HEMK1rc | BNF: GTGTAGGGAGGTAAGGYGTTGTAG BNR: AAATCCRCCCTCCCTACAAACCC | 5 |
|  | F: TTCGTCGTGGGTTTCGGYTTT R: TARCCAARCCCGCTACTA |  |
|  | Probe: CGTTCGCGGATGCGGTTGGTG |  |
|  |  |  |
| HOXA7 | BNF: GAGYTGGTGTTTAAAGTAGAATTTGTT BNR: CAATACRTCCTACCAAAACCTCC | 1 |
|  | F: GTTCGAGTAGGGAGCGATTTC R: ACCTCCTAAACGCGCATCGAA |  |
|  | Probe: ATCGCGGGAAGGAACGTATTTCG |  |
|  |  |  |
| HOXB5 | BNF: GGTGGYTATGGGTTTTGGTTGYGTT BNR: CCAAACCCTCTACTTCRTCCCCCT | 1 |
|  | F: GTTGCGTTCGAGYTAGGTT R1: CCAACGCCAATTTCACCGAAA R2:CCGACCAAACGACCTCAA |  |
|  | Probe: TCGAGGACGCGTTGGYTTCG |  |
|  |  |  |
| HOXB5rc | BNF: GATTAGGYGATTTTAGTTAGYTTTAG BNR: CCATAAACTCTAACTACRCCC | 6 |
|  | F: GYTTTAGCGTTAATTTTATCGAAATAGAC R: CTACGCCCGAGCTAAACTA |  |
|  | Probe: CTCCGAAAACGCGCTARCCTCG |  |
|  |  |  |
| HOXD3a | BNF: GAGGYGTTAGGGTGTTAGG BNR: CAACRACCATTATTCCTAATAAACACA | 5 |
|  | F: GGTGTACGCGGGTTGTTT R: AACGACCAAACAACCTCGRCC |  |
|  | Probe: TGGCGGACGCGTTTGGCGTAC |  |
|  |  |  |
| HOXD3b | BNF: GAGGYTATTAYGGGTAGTAGATT BNR: CTCTTACACCCTACRAACAACA | 1 |
|  | F: GTAGATTTTTGGTTTTTAGCGAGGTTG R: AACCGACGCCTACTTCTAC |  |
|  | Probe: TCGAAAACGAAACGAACGCGCGCA |  |
|  |  |  |
| HOXD9 | BNF: AGAGAGGYGAGGGGAGAATAG BNR: CTAACTCTCCARCTTCCRAACC | 1 |
|  | F: CGTTAGGGGYCGTTTCGAG R: GCGAACGACGAATCCAAAACAC |  |
|  | Probe: TCGGGCGATATCGGYTTGGCG |  |
|  | CF: GTTAGGGGYYGTTTYGAG CR: CRAACRACRAATCCAAAACAC |  |
|  |  |  |
| HOXD10 | BNF: GGTTGGGYTTAGGGGYTGAAG BNR: CACRAACAACAACRACATCTACTAC | 1 |
|  | F: CGGGTTGGYCGAGCGA R: CCCGCGATAATACGCGCTAA |  |
|  | Probe: CGGAGAGCGTTGGGYCGGTTG |  |
|  |  |  |
| FOXH1(KIFC2) | BNF: GGGTGTTTTGGAGATTTYGTAGGG BNR: CCATAAACCAACRCARCTCCC | 5 |
|  | F: TGCGGTGTTTGGAGCGCG R: CTCCCGCTCGCATACC |  |
|  | Probe: TCGCGCGTAGCGTTAGCGTGATT |  |
|  |  |  |
| FOXH1rc | BNF: GGTGTTGTTGTTGTAGGTGGG BNR: CCCCARCTCCACTTAACCCACTC | 6 |
|  | F: CGTGTCGGTCGTCGTTTATTC R: AACCCACTCGATCGACGAACTT |  |
|  | Probe: TACGCGGYCGGAGGATTTCGG |  |
|  |  |  |
| MOXD1 | BNF: TTTGGAGGTAGGGYYGTTYGTGG BNR: CAACRAAACAACCAAATCRCCTTCC | 5 |
|  | F: GTCGGCGGACGTTATGGYT R: CCGCCTCCAAATACGCACTAC |  |
|  | Probe: TGGGCGAGAAGTCGAAGTTTACGTA |  |
|  |  |  |
| NEUROG3 | BNF: GTAGAGGYGGAAGAGGGAGG BNR: CRCTACTTACTCAATACCAACTC | 4 |
|  | F: TTGTCGAGGGGYTTCGAGGA R: GCTCTTAARCCGACTACGTCCC |  |
|  | Probe: AGYTTCGGGTACGGCGCGGG |  |
|  |  |  |
| NODAL | BNF: TTTGTAGGGGAGTGGGTGTTAAGG BNR: AAACTCCCTCTCCRCAACACCCA | 1 |
|  | F: ACGGTAGGGATTTGAGG R: CCTACTCGCCGCGCTAAATACC |  |
|  | Probe: TTTCGAGGATTTGGTAGCGCGTTT |  |
|  |  |  |
| NODALrc | BNF: TGTAGGTTGYGGATGATGTTTGTT BNR: ACRCCTAATAARCCCTACTCCAAAC | 4 |
|  | F: CGCGGTAGAGGTTTAGTATG R: ACTCCAAACGAATACTACGACGA |  |
|  | Probe: TCGCGTACGTAGGAGCGTAGTGG |  |
|  |  |  |
| RASSF5 | BNF: GGATAGYTTTGTTAGTTTTTGGAGG BNR: TACTCCTTARCCCTTCCTCTCTCC | 4 |
|  | F: ATTCGGCGGTTATTTACGG R: GAARCTCGACGCGAATCCGAAA |  |
|  | Probe: AAGCGCGGTTGCGGGAGCG |  |
|  |  |  |
| RASSF5rc | BNF: GGGYYGTTTAGGTTTTGTAGATAG BNR: CTACCAAARCTACCRCCACTAACC | 6 |
|  | F: CGGCGGTTCGGGGTTTAA R: ACCGAACATARCCATAACGTCC |  |
|  | Probe: AGCGGGTACGGGCGTTGTTCG |  |
| NSD1 | BNF: GTTTGGGTGTYGAGAATTGTAG BNR: AATCCTCTAATCCCTACACCRCC | 6 |
|  | CF: CGCGGYTCGTTTCGTTTCGG CR: CGCGCCTAATCAAAATCTAARCC |  |
